# Collective cell migration of *Dictyostelium* without cAMP oscillations at multicellular stages

**DOI:** 10.1038/s42003-018-0273-6

**Published:** 2019-01-24

**Authors:** Hidenori Hashimura, Yusuke V. Morimoto, Masato Yasui, Masahiro Ueda

**Affiliations:** 1 Department of Biological Sciences, Graduate School of Science, Osaka University, Suita, Osaka, 565-0871 Japan; 2 RIKEN Center for Biosystems Dynamics Research (BDR), Suita, Osaka, 565-0874 Japan; 3 Department of Bioscience and Bioinformatics, Faculty of Computer Science and Systems Engineering, Kyushu Institute of Technology, Iizuka, Fukuoka, 820-8502 Japan; 4 Graduate School of Frontier Biosciences, Osaka University, Suita, Osaka, 565-0871 Japan

## Abstract

In *Dictyostelium discoideum*, a model organism for the study of collective cell migration, extracellular cyclic adenosine 3’,5’-monophosphate (cAMP) acts as a diffusible chemical guidance cue for cell aggregation, which has been thought to be important in multicellular morphogenesis. Here we revealed that the dynamics of cAMP-mediated signaling showed a transition from propagating waves to steady state during cell development. Live-cell imaging of cytosolic cAMP levels revealed that their oscillation and propagation in cell populations were obvious for cell aggregation and mound formation stages, but they gradually disappeared when multicellular slugs started to migrate. A similar transition of signaling dynamics occurred with phosphatidylinositol 3,4,5-trisphosphate signaling, which is upstream of the cAMP signal pathway. This transition was programmed with concomitant developmental progression. We propose a new model in which cAMP oscillation and propagation between cells, which are important at the unicellular stage, are unessential for collective cell migration at the multicellular stage.

## Abstract (web summary)

Hidenori Hashimura et al. show that cAMP-mediated signaling changes from propagating waves to a steady state at a multicellular stage of amoeba *Dictyostelium discoideum*. This study suggests that cAMP oscillation is not required for collective cell migration despite its importance at a unicellular stage.

## Introduction

Collective cell migration is ubiquitous in multicellular organisms and contributes to many organismal phenomena, including morphogenesis, wound healing, and cancer invasion^1,2^. It is organized by integrated physical and chemical guidance cues between cells, such as cell–cell adhesion and diffusible factor-mediated signaling, which are integrated and act in parallel^3^. The social amoeba *Dictyostelium discoideum* is a model organism for the study of collective cell migration because of its morphogenesis and simple cell–cell interactions via diffusible chemical signals^1,4^. *Dictyostelium* cells grow as unicellular organisms at the vegetative stage, but undergo transitions from a unicellular to multicellular organism by aggregation upon starvation. During aggregation, starved cells typically move towards the aggregation center to form one multicellular aggregate. This coordinated migration is achieved by the self-organization of cAMP gradients and by chemotaxis to extracellular cAMP^5^. When *Dictyostelium* cells sense extracellular cAMP signals, cAMP receptors activate PI3-kinases through G proteins to produce phosphatidylinositol 3,4,5-trisphosphate (PIP3) transiently on the plasma membrane of the cell front, leading to the transient localization of cytosolic regulator of adenylyl cyclase (CRAC) to the membrane via the Pleckstin Homolog (PH) domain that binds to PIP3, activating adenylyl cyclase^6,7^. The *Dictyostelium* cell has three subtypes of adenylyl cyclase (ACA, ACB, and ACG), but only ACA is activated by external cAMP signals^8^. cAMP is synthesized by ACA in response to external cAMP signals and secreted to induce neighboring cells to similarly produce cAMP. Simultaneously, the transient accumulation of PIP3 at the cell front in response to external cAMP also induces actin polymerization and pseudopod formation, resulting in chemotactic migration^9^. These reactions finally cause the propagation of cAMP signals as travelling waves called cAMP relay, resulting in chemotactic migration toward the aggregation center. That is, the correlative migrations of multiple cells are mediated by a single diffusible chemical factor, extracellular cAMP.

It has been argued that cAMP relay is also essential for the organization of collective cell migration during developmental events following the aggregation^10^. Upon aggregation, cells form a stream which flows into a loose mound. Loose mounds become tightly packed (tight mounds) by both secretion of the extracellular matrix and the strengthening of cell–cell contacts. In tight mounds, cells differentiate into prestalk or prespore cells. Prestalk cells are sorted at the top of the mound to form the tip, which elongates and forms the front of a multicellular body (slug) to migrate as a whole. In conventional microscopic observations, optical densities of cell populations during chemotactic aggregation describe synchronous changes in cell shapes and act as an index of cAMP relay^11^. These optical density waves have been detected in streams, mounds, and slugs, giving evidence of cAMP relay at these stages too^12,13^. Cell sorting to the tip of the mound also can be explained by cAMP relay. There is a difference in the response of chemotaxis toward cAMP between prespore and prestalk cells in mounds, resulting in cAMP relay guiding the sorting of prestalk cells to the tip of the mound^14,15^. Cells dissociated from slugs produce cAMP upon extracellular cAMP stimulation^16^ and show chemotactic movement toward cAMP^17^, indicating that slug cells have the ability of cAMP relay and chemotaxis toward cAMP. Furthermore, cAMP microinjection in slugs causes chemotactic attraction of some cells in the population and perturbation of the optical density wave propagation^13,18^. These observations suggest that cAMP signals control cell movement in slugs. Thus, cAMP relay is regarded as an essential mechanism for organized collective cell migration, such as cell sorting and multicellular movement, in *Dictyostelium* cells.

In spite of these traditional views of cAMP relay for the coordination of collective cell migration in *Dictyostelium*, some observations suggest that the role of cAMP relay in slugs is controversial. *acaA*-null cells, which lack the ability of cAMP relay, normally cannot aggregate and form multicellular bodies, but the phenotypes of the mutant are rescued by constitutive activation of PKA, which is downstream of the cAMP signaling pathway, implying that *Dictyostelium* cells have developmental ability without cAMP oscillation^19^. Furthermore, cAMP signals in mounds and slugs have not been investigated, whereas the cAMP relay during cell aggregation has been directly verified by live imaging of cAMP signals using sophisticated cAMP-sensitive fluorescent probes, which has revealed that intracellular and extracellular cAMP levels show synchronous oscillations in cell populations and that propagation of the oscillations changes between cells^20,21^. Therefore, no clear evidence exists for cAMP relay organizing collective cell migration at multicellular stages. In this study, we investigated the dynamics of cAMP signals through the development of *Dictyostelium* cells by visualizing the changes in cytosolic cAMP levels ([cAMP]_i_), which reflect the response to cAMP relay. Our live-imaging approaches demonstrated the role of cAMP relay during aggregation and mound stages. Surprisingly, we found that [cAMP]_i_ oscillation and its propagation, which is an index of cAMP relay, gradually decreased and disappeared after slug formation. This result indicates a dramatic transition of cAMP signaling dynamics during the development of *Dictyostelium* cells and the possibility that oscillatory cAMP signaling is not essential for collective cell migration in slugs, which challenges the traditional view about the role of cAMP relay in the organization of collective cell migration.

## Results

### Flamindo2 is an indicator of cytosolic cAMP levels in *Dictyostelium* cells

To investigate cAMP relay in collective cell migration during the development of *Dictyostelium* cells, we monitored [cAMP]_i_ by using a cAMP indicator, Flamindo2^22^. The binding of cAMP to the probe causes a decrease in the fluorescence intensity of the sensor. It has been reported that Flamindo2 can detect [cAMP]_i_ changes in aggregating *Dictyostelium* cell populations^23^. We confirmed that Flamindo2 was stably expressed in *Dictyostelium* cells with no obvious defects in the developmental progression (Supplementary Fig. 1a, b). The fluorescence intensity of the sensor in the cytosol of chemotactic-competent cells showed transient changes with two peaks after external cAMP stimulation; the first peak occurred 15 s after the stimulation, and the second peak gradually appeared 120 s after (Supplementary Fig. 2a, first panel). In *acaA*-null cells, the first peak of the response was weaker than in wild-type cells, and the second peak had completely disappeared (Supplementary Fig. 2a, second panel). When wild-type cells were treated with 4 mM caffeine, which inhibits adenylyl cyclase activities^24^, the second peak of the fluorescence intensity after the cAMP stimulation had again disappeared (Supplementary Fig. 2a, third panel). It has been reported that cytosolic cAMP and cGMP levels show different response times to cAMP stimulation and that the first response of [cGMP]_i_ elevation occurs within 10 s of the stimulation, but the [cAMP]_i_ elevation occurs later (second peak)^25^. It has been shown using biochemical assays and FRET-based imaging analyses that the peak of ACA activity occurs 60–120 s after external cAMP stimulation^26,27^. Considering Flamindo2 binds not only to cAMP but also to cGMP but with lower affinity^22^, our results suggest that the first peak is an effect of [cGMP]_i_ elevation and that the second peak was due to only the increase in [cAMP]_i_. This conclusion is also supported by *gc-* cells, which lack guanylyl cyclases (*gca* and *sgcA*) and have no cGMP production ability^28^, showing Flamindo2 signal responses with only one peak at 90 s after the stimulation (Supplementary Fig. 2a, fourth panel). When cells expressing only Citrine instead of Flamindo2 in the cytosol were stimulated with cAMP, the fluorescence intensity showed no response (Supplementary Fig. 2b), indicating that changes in the Flamindo2 signal were not caused by cell deformation or other signals. Finally, the response was dose-dependent to the concentration of the applied extracellular cAMP (EC_50_, 0.72 nM; Supplementary Fig. 2c), which is in good agreement with a previous report^20^.

To see whether Flamindo2 is applicable to the monitoring of [cAMP]_i_ during the development of *Dictyostelium* cells, we examined cell populations in the early aggregation stage, which is the event achieved by cAMP relay and chemotaxis^5,20^. Flamindo2 signals showed obvious synchronous oscillations during early aggregation, and the pharmacological inhibition of ACA by caffeine treatment caused severe defects in the oscillations (Supplementary Fig. 2d). Such synchronous oscillations of Flamindo2 signals also could be detected in aggregating *gc*- cells (Supplementary Fig. 2e). As expected, *acaA*-null cells did not aggregate (Supplementary Fig. 2f) and showed no obvious oscillations of Flamindo2 signals after starvation (Supplementary Fig. 2g). These results demonstrate successful monitoring of ACA-dependent cAMP relay at the early aggregation stage by using Flamindo2^23^. Because the protein levels of Flamindo2 in slugs were less than those in aggregating cells (ca. 62%; Supplementary Figure 3a), we assessed whether the protein expression levels affect the sensitivity of Flamindo2. Based on fluorescence intensities of Flamindo2, we found variation in the expression levels (Supplementary Figure 3b), which we classified into three levels: low, moderate, and high. The expression levels in the low group was ~30% than that in the moderate group. No groups showed different sensitivities to different external cAMP concentrations (Supplementary Fig. 3c–f). Therefore, the sensitivity of Flamindo2 was independent of the expression level variation.

### Transition of cAMP signaling dynamics from oscillations to steady state during *Dictyostelium* development

**Fig. 1:**
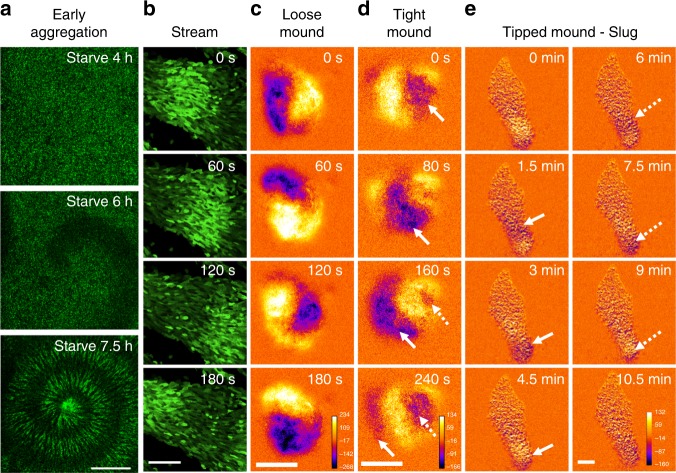
Typical cAMP signaling dynamics at each developmental stage of *Dictyostelium* cells visualized by Flamindo2. **a** Spiral pattern of a [cAMP]_i_ wave in cell populations at early aggregation. **b** Wave propagation in an aggregating stream. **c** Rotational propagation in a loose mound. **d** Wave propagation from the top of a tight mound (right side of images) to the bottom. **e** A slug with a stream elongating toward the top of the images. In **c**–**e**, images were subtracted at 3–6 frame intervals to emphasize changes in fluorescence intensity. Solid and broken arrows show the positions of the first and second waves in each sequential image, respectively. Scale bars, **a** 1 mm, **b**, **e** 100 μm, **c**, **d** 50 μm

Using Flamindo2, we monitored the dynamics of cAMP relay during the development of *Dictyostelium* cells. Figure 1a, b and Supplementary Movie 1 show the propagation of [cAMP]_i_ waves during early aggregation and streams. In the loose mound stage, [cAMP]_i_ waves showed rotational propagation (Fig. 1c and Supplementary Movie 2). At the tight mound stage, [cAMP]_i_ waves exhibited propagation from the top to the bottom of the mound with geometrical changes (Fig. 1d and Supplementary Movie 2). In contrast, subsequent mound elongation and slug migration occurred without obvious [cAMP]_i_ oscillations, but a stream flowing into the rear of the mound showed wave propagation (Fig. 1e and Supplementary Movie 3). These findings indicate the dynamics of cAMP signaling changes from propagating waves in aggregation, streams, and mounds to steady state with no oscillations in migrating slugs during *Dictyostelium* development.

**Fig. 2:**
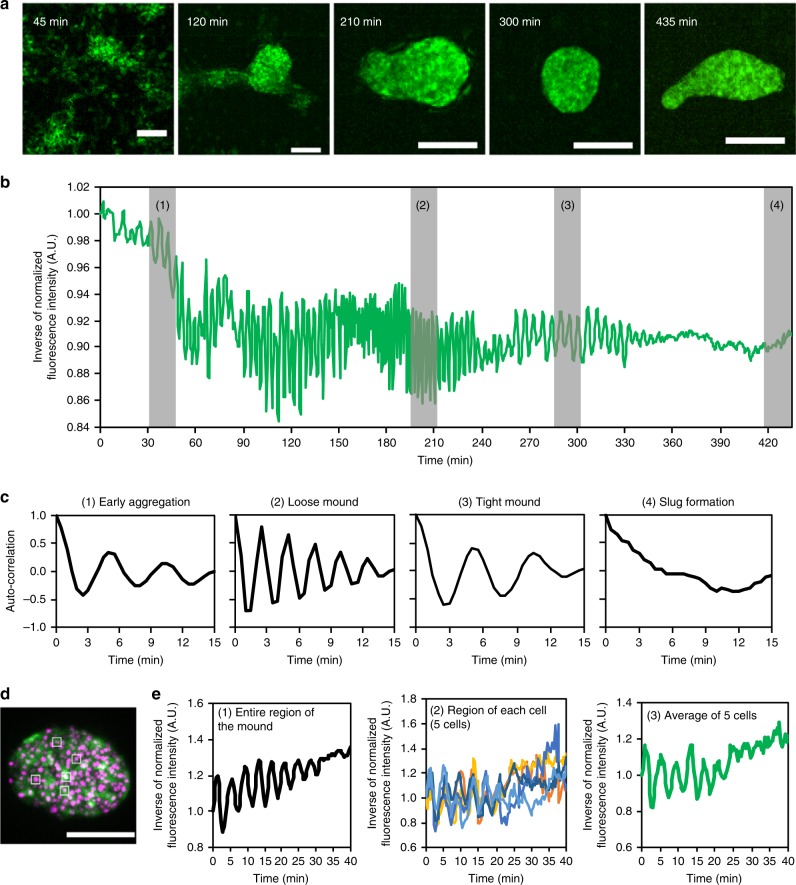
Disappearance of [cAMP]_i_ oscillations during development. **a** Fluorescent images of *Dictyostelium* cells expressing Flamindo2 in each developmental stage. Maximum intensity projections of Z-stack images are shown. Scale bars, 100 μm. **b** Time course plot of inverse Flamindo2 signals during development from the onset of aggregation to slug formation. Data were obtained 3.5–10.75 h after starvation. The mean intensity of Flamindo2 in a 30 μm^2^ region in the cell population shown in **a** was measured. **c** Autocorrelation of Flamindo2 signals at each development stage are shown by the gray bars in **b**. **d** A fluorescence image of Flamindo2 and Histone2B-RFP in an elongating mound. The maximum intensity projection of Z-stacks is shown. Scale bar, 50 μm. **e** Time course plot of inverse Flamindo2 signals at the tissue (first) or individual cell level (second and third) in the mound shown in **d**. First, average signals in the entire region of the mound. Second, signals in 5 cells indicated by the white boxes in **d**. In the second graph, individual cells were tracked, and Flamindo2 intensities within each cell were measured. Third, average of the signals in the second graph

**Fig. 3:**
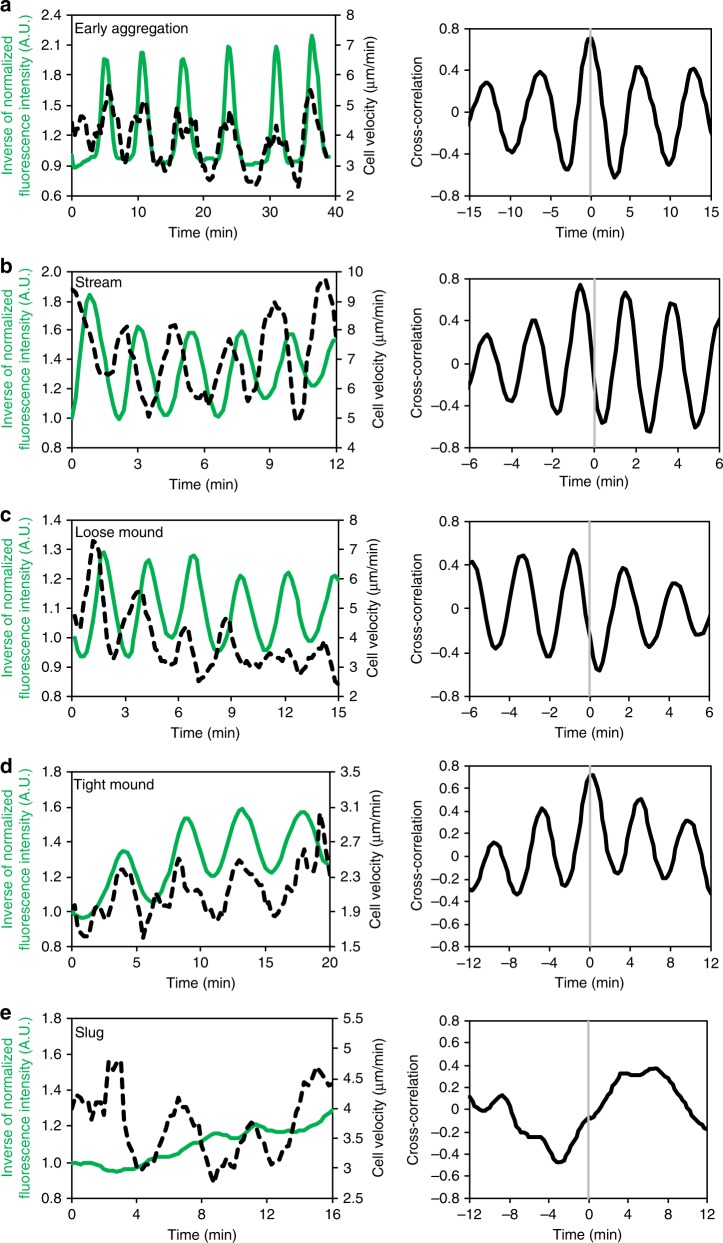
Simultaneous monitoring of [cAMP]_i_ and cell velocity at each developmental stage. Left graphs show time-course plots of [cAMP]_i_ (green solid lines) and cell velocity (black dashed lines). Individual cells were tracked, and Flamindo2 intensities within each cell and cell velocities were measured. The signals of Flamindo2 and cell velocities were averaged across several representative cells, and the averages of representative cells are plotted against time. The curves of Flamindo2 signals and cell velocities were smoothed by a running average over four data points. Right graphs show the cross-correlation between [cAMP]_i_ and cell velocity shown in the left graphs. **a** Early aggregation (*n* = 20 cells). **b** Aggregation stream (*n* = 14 cells). **c** Loose mound (*n* = 12 cells). **d** Tight mound (*n* = 10 cells). **e** Slug (*n* = 10 cells)

To characterize the transition of the cAMP signaling dynamics, we analyzed Flamindo2 signals from the onset of aggregation to the slug stage (Fig. 2a, b and Supplementary Movie 4). During early aggregation, synchronized oscillations of [cAMP]_i_ started with periods of 5.62 ± 0.36 min (Fig. 2b, c and Table 1). Such oscillations continued until mound formation. At these points, the periods became shorter in loose mounds (2.47 ± 0.28 min), but showed partial recovery in tight mounds (4.70 ± 0.56 min) (Fig. 2b, c and Table 1). Simultaneous monitoring of Flamindo2 signals and cell movements revealed that the cell velocity oscillated with the same period as [cAMP]_i_ (Table 1) and that the two oscillations had tight correlation with each other (Fig. 3a–d). [cAMP]_i_ oscillations in the cell populations had the same intervals and were synchronized at the individual cell level, while the oscillations of the cell velocity showed some variation in the populations (Supplementary Fig. 4, 5). Cross-correlation analysis indicated that there was a phase difference of a half period between the oscillation of [cAMP]_i_ and cell velocity at the loose mound stage, but the two oscillations had the same phase at the early aggregation and tight mound stages (Table 1). These findings indicate that oscillatory cAMP signaling organizes collective cell migration until the mound formation.

**Table 1:** Periods of oscillation of [cAMP]_i_ and cell velocity at three developmental stages

	Early aggregation	Loose mound	Tight mound	Slug
Periods of cell velocity (min)	5.58 ± 0.47	2.48 ± 0.22	4.78 ± 0.31	5.32 ± 1.09
Periods of [cAMP]_i_ oscillation (min)	5.62 ± 0.36	2.47 ± 0.28	4.70 ± 0.56	ND
Phase difference between cell velocity and [cAMP]_i_ oscillation (min)	0.29 ± 0.29	1.26 ± 0.26	0.15 ± 0.12	ND

Mean values ± SD are shown. Sample numbers are as follows: 100 cells in 6 aggregation centers (early aggregation), 45 cells in 7 mounds (loose mound stage), 47 cells in 5 mounds (tight mound stage), and 20 cells in 11 slugs (slug stage). The periods of [cAMP]_i_ oscillations and cell velocities in the loose mound stage are significantly shorter than those in the early aggregation and the tight mound stages (*P* *<* 10^–5^: Student’s two-tailed *t*-test)

*ND* no detection of periodicity

We next examined the [cAMP]_i_ dynamics during slug formation and migration. When slugs were formed through mound elongation and tip formation, [cAMP]_i_ oscillations became weaker and finally disappeared (Fig. 2b, c). To see whether the apparent disappearance of the oscillations in multicellular bodies resulted from a desynchronization of oscillations between cells or a synchronous disappearance of the oscillations, [cAMP]_i_ dynamics during the damping of oscillations in an elongating mound was monitored at the single cell level (Fig. 2d). When the oscillation of Flamindo2 signals in the entire mound had almost disappeared (Fig. 2e, left: ~25 min), oscillation in single cells vanished almost at the same time (Fig. 2e, middle and right). Thus, the disappearance of the [cAMP]_i_ oscillations in the entire mound was caused by a synchronous disappearance of oscillations in individual cells. The Flamindo2 signals of 24 migrating slugs for 20 min were observed, but no slugs showed [cAMP]_i_ oscillations. We also measured [cAMP]_i_ and cell velocity of the cells in slugs. Prestalk cells made up ~20% of slugs (anterior) and moved rotationally, while prespore cells made up ~80% of slugs (posterior) and moved straight (Supplementary Fig. 6a, b). Cell movements in the anterior and posterior (respectively the prestalk and prespore regions) showed oscillations with periods of 7.75 and 8.25 min. However, no obvious oscillations in [cAMP]_i_ associated with cell velocity were observed (Fig. 3e, Supplementary Fig. 6c, d). These results suggest that the dynamics of cAMP relay changes after slug formation and that the collective cell migration in slugs does not depend on oscillatory cAMP signaling.

### Verification Flamindo2 functions as a cAMP indicator in slugs

**Fig. 4:**
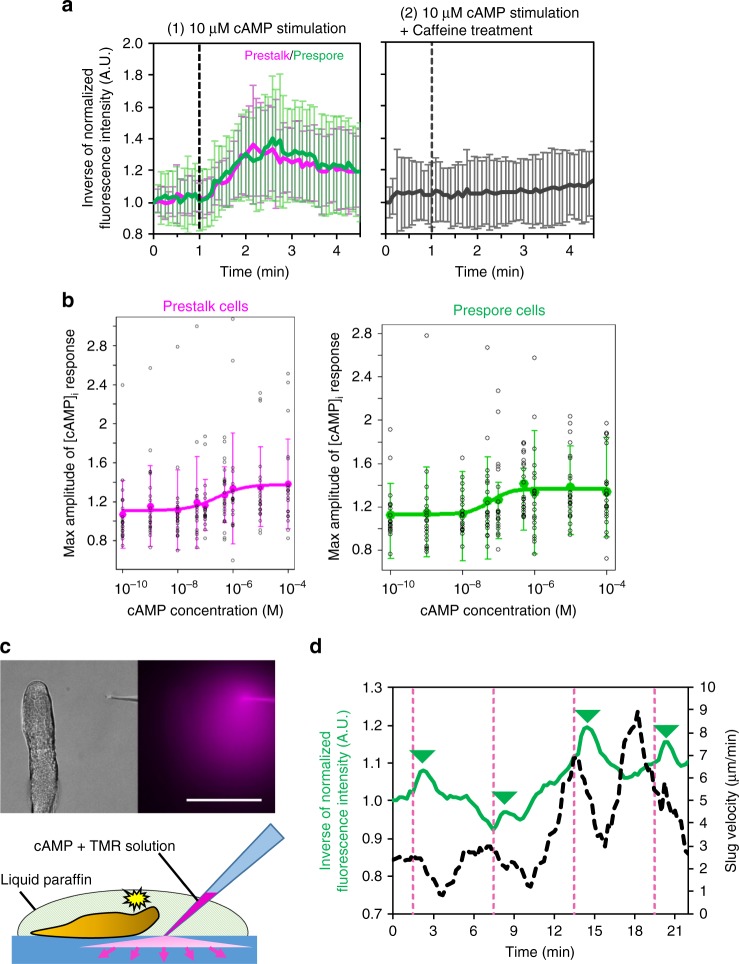
[cAMP]_i_ response of slug cells to external cAMP stimulation. **a** Time course plot of inverse Flamindo2 signals in slug-dissociated cells after 10 μM cAMP stimulation (mean ± SD). Left, no-treatment cells (magenta, prestalk cells, *n* = 22 cells; green, prespore cells, *n* = 22 cells). Right, caffeine-treated cells (*n* = 45). **b** A dose-dependent curve of [cAMP]_i_ response to various concentrations of cAMP stimuli (0.01–10 μM, mean ± SD). Magenta, prestalk cells (*n* = 20–24 cells at each data point). Green, prespore cells (*n* = 20–22 cells at each data point). **c** External cAMP stimulation to the slug by injection of cAMP into agar near the slug from a microcapillary. Left, DIC image. Right, fluorescent image of diffusing dye mixed with cAMP to visualize the injected solution. Scale bar, 100 μm. Bottom, a scheme of the cAMP microinjection experiment. cAMP solution mixed with the dye is diffused from the tip of a micropipette into agar to stimulate the entire slug. **d** Time-course plot of inverse Flamindo2 signals in whole slug (green solid line) and slug velocity (black dashed line). Dashed magenta lines indicate time of the cAMP injection. The mean intensity of Flamindo2 in a 43 × 186 μm^2^ region in the slug shown in **c** was measured. The curves of slug velocity were smoothed by a running average over six data points. The peaks of Flamindo2 signals after cAMP stimulation are shown as green triangles

To see whether the absence of [cAMP]_i_ oscillations was due to the loss of Flamindo2 function in slugs, we stimulated cells dissociated from the slugs with external cAMP and monitored [cAMP]_i_. Prestalk and prespore cells showed similar [cAMP]_i_ responses to external cAMP stimulation (Fig. 4a, first), which is consistent with the results obtained from previous biochemical assays^16^. The responses were completely suppressed when the cells were treated with caffeine (Fig. 4a, second). The dose-dependency of the response showed an EC_50_ of 250 ± 136 nM and 58 ± 22 nM in the prestalk and prespore cells, respectively (Fig. 4b), values that are ~100-fold higher than in the unicellular phase (Supplementary Fig. 2c). The difference of EC_50_ between prestalk and prespore cells was not significant. These findings verified Flamindo2 was functional in slug cells. We next examined the response of intact slugs by using a micropipette containing cAMP solution. The response of slugs to cAMP stimulation has been investigated by the direct injection of cAMP solution into slugs from a micropipette in previous studies^13,18^, but we stimulated slugs by injecting cAMP from the micropipette into agar to diffuse it and avoid mechanical stimulation through contact between the micropipette and slugs (Fig. 4c and Supplementary Movie 5). This application of cAMP to a slug caused transient changes in the Flamindo2 signals and slug velocity (Fig. 4d). Thus, extracellular cAMP signals could modify slug movements, as reported previously^13^, and induce cAMP production, although endogenous [cAMP]_i_ waves were not detected in slugs.

### Transition of PIP3 signaling dynamics with progression of *Dictyostelium* development

It has been reported that the translocation of PH_CRAC_-GFP to the leading edge of cells, which indicates a transient increase of PIP3 levels on the plasma membrane in response to cAMP signals, is periodic in mounds but becomes non-periodic in slugs^29^. To confirm whether such a transition is observed also with our observation system and whether the PIP3 signal dynamics such as oscillation periods at each developmental stage corresponds to the [cAMP]_i_ dynamics, we investigated the dynamics of PIP3 signaling using GFP fused to the PH domain of Akt/PKB. The PH domain of Akt/PKB is reported to show transient localization to the plasma membrane in response to external cAMP stimulation dependently on PI3-kinase activity^30,31^. We confirmed that cells dissociated from loose mounds or slugs show a transient translocation of PH_Akt/PKB_-GFP to the plasma membrane in response to uniform cAMP stimulation and found that the adaptation in slug-dissociated cells was imperfect (Supplementary Fig 7a–c), although the previous study using PH_CRAC_-GFP did not succeed in detecting the transient elevation of PIP3 levels on the plasma membrane of slug-dissociated cells^29^. Loose and tight mounds exhibited periodic translocations of PH_Akt/PKB_-GFP to the leading edge of individual cells and PH_Akt/PKB_-GFP propagation between cells (Supplementary Fig. 8a–d and Supplementary Movie 6). In slugs, however, PH_Akt/PKB_-GFP was continuously localized at the leading edge of the cells and did not show any periodic translocation to the membrane (44 cells, 15 slugs; Supplementary Fig. 8a–d and Supplementary Movie 6). The periods of translocation in loose and tight mounds were 2.89 ± 0.95 min (25 cells, 4 mounds) and 5.17 ± 1.32 min (20 cells, 4 mounds), respectively, which agrees well with the periods of [cAMP]_i_ oscillations (Table 1). Furthermore, to see whether continuous localization of PH_Akt/PKB_-GFP in slug cells depended on cAMP signals or not, interference on the localization of PH_Akt/PKB_-GFP in intact slugs by cAMP stimulation was investigated by the cAMP microinjection assay. cAMP stimulation did not affect the localization of PH_Akt/PKB_-GFP of cells in an intact slug (Supplementary Fig. 7d). Additionally, the interference of cAMP signaling by caffeine did not affect the localization of PH_Akt/PKB_-GFP in slug cells (Supplementary Fig. 7e). These findings are consistent with previous results^29^ and suggest that the continuous localization of PH_Akt/PKB_-GFP to the leading edge of cells in slugs does not depend on cAMP signals. Rather, the results suggest that the cAMP signaling pathway upstream of cAMP production undergoes a transition in its dynamics during slug formation.

### Development of *acaA-*null cells without [cAMP]_i_ oscillations

**Fig. 5:**
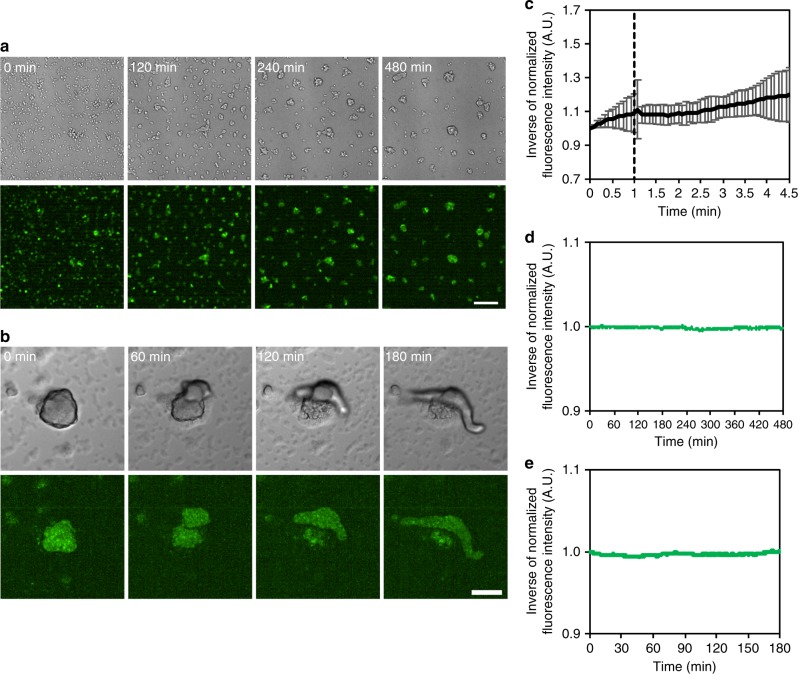
Development of *acaA*-null cells without [cAMP]_i_ oscillations. **a** Aggregation of *acaA*-null cells expressing Flamindo2 in DB with exogenous cAMP pulses under microscopic observation. Top panels, DIC images. Lower panels, fluorescent images of Flamindo2. Scale bar, 100 μm. **b** Slug formation of *acaA*-null cells expressing Flamindo2 on agar. Cells were washed and deposited on an agar plate after cAMP pulses. Upper panels, DIC images. Lower panels, fluorescent images of Flamindo2. Inside the fluorescent images, maximum intensity projections of Z-stacks are shown. Scale bar, 100 μm. **c** Time-course plot of Flamindo2 signals in cell clumps after 100 μM cAMP stimulation. The mean intensity of Flamindo2 in a 25 μm^2^ region in the cell mass was measured, and the inverse of the fluorescence intensity of Flamindo2 is plotted on the y-axis (mean ± SD, *n* = 13 clumps). **d** Time-course plot of Flamindo2 signals in *acaA*-null cells during aggregation. The mean intensity of Flamindo2 in a 100 μm^2^ region on the aggregation field shown in **a** was measured, and the inverse of the fluorescence intensity was plotted against time. **e** Time-course plot of Flamindo2 signals in *acaA*-null cells during slug formation. The mean intensity of Flamindo2 in a 100 μm^2^ region in the cell mass shown in **b** was measured, and the inverse of the fluorescence intensity was plotted against time

Our hypothesis that cAMP relay is dispensable for the collective cell migration of multicellular slugs is incompatible with the model that assumes cAMP relay plays key roles in organized collective cell migration in slugs. However, it is consistent with the fact that *acaA-*null cells lacking cAMP relay can aggregate and develop to form multicellular bodies when the expression of developmental genes is induced by exogenous and uniform cAMP pulses^32^. To confirm whether *acaA*-null cells could develop and migrate as multicellular organisms without [cAMP]_i_ oscillations, we monitored Flamindo2 signals during their development. When *acaA*-null cells were exposed to exogenous cAMP pulses, small clumps were formed by aggregation (Fig. 5a and Supplementary Movie 7). After terminating the exogenous cAMP pulses, the clumps deposited on agar started to elongate and then formed migrating slugs (Fig. 5b and Supplementary Movie 8). We found that the Flamindo2 signals from cell clumps formed by aggregation were unresponsive to external cAMP stimulation (Fig. 5c), and no obvious [cAMP]_i_ oscillations were observed during the aggregation or slug stages (Fig. 5d, e). These observations suggest that oscillatory cAMP signaling is not essential for collective cell movements in migrating slugs.

### Transition of cAMP signaling dynamics occurs with the progression of development

**Fig. 6:**
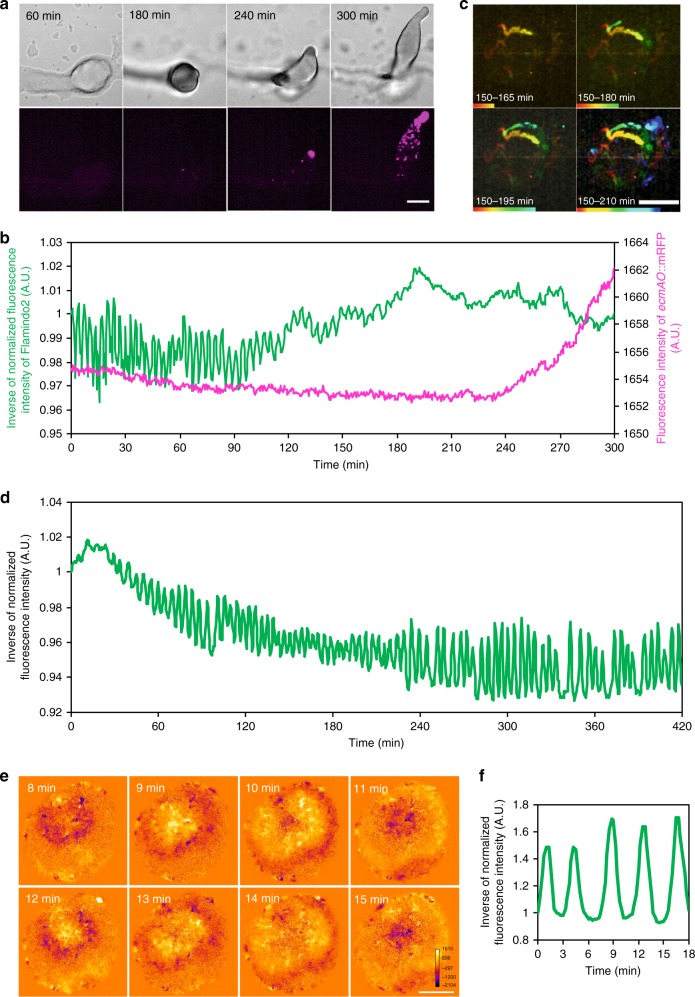
Association of cAMP signaling transition with developmental progression. **a** Expression of *ecmAO*::mRFPmars during mound development. Upper panels, DIC images. Lower panels, fluorescent images of *ecmAO*::mRFPmars. Scale bar, 100 μm. **b** Time course plot of Flamindo2 (green) and *ecmAO*::mRFPmars (magenta) signals during mound development. Data were obtained 8–13 h after starvation. The mean intensity of Flamindo2 in a 30 μm^2^ region in the mound shown in **a** and the mean intensity of *ecmAO*::mRFPmars in the entire region of the mound were measured. **c** The sorting of cells expressing *ecmAO*::mRFPmars at the top of the tight mound. This figure shows trajectories of sorted prestalk cells marked by the expression of *ecmAO*::mRFPmars at the top of the mound (upper right side of images) during the mound elongation. Scale bars, 100 μm. **d** Time course plot of inverse Flamindo2 signals during the development of *gbfA*^−^ cells. Data were obtained 3–10 h after starvation. The mean intensity of Flamindo2 in a 50 μm^2^ region of the cell population was measured. **e** Wave propagation of Flamindo2 signals in the loose mound of *gbfA*^−^ cells after 24 h starvation. Images were subtracted at 4 frame intervals to emphasize changes in the signals. Scale bar, 50 μm. **f** Time course plot of inverse Flamindo2 signals in the mound shown in **e**. The mean intensity of Flamindo2 in a 20 μm^2^ region in the mound shown in **e** was measured

To reveal the temporal relationship between the transition of cAMP dynamics and development, we observed cAMP dynamics and tip formation simultaneously using Flamindo2 and *ecmAO*::mRFPmars (Fig. 6a and Supplementary Movie 9), because the tip of the mound is regarded as a prestalk region characterized by the high expression of the *ecmAO* gene^33^. [cAMP]_i_ oscillated clearly from the loose to tight mound stage with no obvious expression of mRFPmars under the control of the *ecmAO* promoter (Fig. 6b, 0–120 min). When the tight mound begun to elongate and cells highly expressing *ecmAO*::mRFPmars were sorted into the tip (Fig. 6c), [cAMP]_i_ oscillations became weaker and finally disappeared upon complete tip formation (Fig. 6b, 120–210 min). Considering the maturation time of mRFP has a time lag of ~90 min^34^, both the accumulation of prestalk cells to the tip and the loss of cAMP waves started to occur around the same time (Fig. 6b, c, ~150 min), suggesting that the transition of the [cAMP]_i_ dynamics occurred simultaneously with the tip formation. To confirm whether the transition of cAMP signaling is a developmentally regulated event, we examined the [cAMP]_i_ dynamics of a mutant lacking *gbfA*, which encodes the transcription factor G-box binding factor (GBF). GBF regulates late-development gene expression, and *gbfA*-null cells undergo developmental arrest at the loose mound stage without tip formation due to the lack of post-aggregative and cell-type specific genes^35^ (Supplementary Fig. 9). The results show that mutant cells had impaired transitions in cAMP dynamics, in which cAMP waves continued over 7 h and their propagation persisted 24 h after starvation with arrest in the loose mound stage (Fig. 6d–f). These results are consistent with a previous report that found mounds of a *gbfA*-null mutant showed optical density waves, although the pattern of the wave propagation was aberrant^36^. Thus, the cAMP dynamics transition from propagating waves to steady state was due to developmental regulation.

## Discussion

In past studies, the details of cAMP signal dynamics have been examined only at the unicellular stage^20,27^. In contrast, our study investigated the dynamics of cAMP signaling throughout the development of *Dictyostelium* cells including the multicellular phase by using the cAMP indicator Flamindo2. Flamindo2 could detect the [cAMP]_i_ changes of *Dictyostelium* cells in response to external cAMP stimuli (Supplementary Fig. 2, Fig. 4), and purified Flamindo2 has an EC_50_ of 3.2 μM to cAMP and Hill coefficient of 0.95^22^, which covers the range of cytosolic cAMP levels measured biochemically in unstimulated and cAMP-stimulated *Dictyostelium* cells at all development stages^8,16,37^. We estimated the cytosolic cAMP concentration to be ~0.3–12 μM, based on the values of cell volume and protein amount in previous reports^38,39^. These estimates indicate that Flamindo2 is an appropriate tool for monitoring the cAMP signaling dynamics in *Dictyostelium* cells throughout their development. In the aggregation and mound stages, we observed [cAMP]_i_ wave oscillations and wave propagations in the cell populations (Figs 1, 2). The wave oscillations were tightly coupled with the cell movement (Fig. 3a–d). The phase relationship between the [cAMP]_i_ oscillation and cell velocity was dependent on the wave periods and not on the developmental stage (Table 1), suggesting that the relationship between cAMP production and chemotactic movement in response to the cAMP signal is maintained until the tight mound stage. The pattern of [cAMP]_i_ wave propagation (Fig. 1a–d) and the [cAMP]_i_ oscillation period in the aggregation and mound stages (Table 1) agreed well with synchronous changes in the optical density of cell populations previously reported^11,12^. The oscillation period decreased in loose mounds, but increased when tight mounds were formed (Table 1). These changes would be caused by two reasons; previous reports suggest that the decrease of the oscillation period can be explained by an increase in the cell density and extracellular cAMP^20,27,40^, while the increase of the oscillation period can be explained by the expression of low-affinity cAMP receptors during the mound stage^41^ instead of high-affinity receptors expressed in the aggregation stage. It has also been suggested that the expression of low-affinity cAMP receptors causes changes in the cAMP wave geometry^41^, which is agreement with the hypothesis that the expression of low-affinity cAMP receptor plays a key role in changing the [cAMP]_i_ wave propagation pattern at the stage from loose mounds to tight mounds (Fig. 1c, d). Furthermore, the changed wave geometry in the mound stages agrees with the classical model, which assumes prestalk cells are sorted on the top of the tight mound by chemotaxis toward cAMP signals^4^. Overall, our findings show collective cell migration was coordinated with cAMP relay from the early aggregation to tight mound stages, which is consistent with the mechanism of collective cell migration in *Dictyostelium* cells^1,4^.

Our observations revealed that [cAMP]_i_ wave oscillations and wave propagations gradually weakened with slug formation and eventually disappeared (Fig. 2b, c), although cell velocity oscillations in slugs were consistent with those in early aggregation and mound stages (Fig. 3e and Supplementary Fig. 6c, d). Because a transient elevation of [cAMP]_i_ in slug cells in response to external cAMP stimuli was observed (Fig. 4), we confirmed the vanishing of the [cAMP]_i_ oscillation was not due to impaired Flamindo2 function. Rather, our observations show that a transition of cAMP signaling dynamics occurs after slug formation and that any endogenous [cAMP]_i_ changes in slugs was below the detection limit of Flamindo2. We confirmed cAMP signaling transitions by investigating the dynamics of PIP3 signaling, which activates adenylyl cyclase and in turn produces cAMP. We monitored PIP3 levels on the plasma membrane using PH_Akt/PKB_-GFP and found periodic changes at the mound stages but not at the slug stage (Supplementary Fig. 8). This contrast is consistent with a previous report that tracked the PH domain of CRAC by GFP labeling^29^. In addition, we found a correlation between the oscillations of [cAMP]_i_ and PH_Akt/PKB_-GFP translocation (Table 1). Although the cells dissociated from slugs showed a transient translocation of PH_AKT/PKB_-GFP in response to cAMP stimuli, the continuous localization of PH_AKT/PKB_-GFP to the leading edge of cells in intact slugs was not inhibited by external cAMP stimuli or caffeine treatment (Supplementary Fig. 7). These observations suggest that the constant polarity of PIP3 levels on the cell membrane of slug cells depends on tonic cAMP signals and/or other signals such as cell–cell contacts, as indicated in a previous study^29^. Thus, our findings demonstrate transitions from oscillations to steady state upstream of the cAMP signaling pathway during slug formation. Further, they raise the possibility that collective cell migration at the slug stage does not depend on oscillatory cAMP signaling for cell–cell communication, which challenges existing models^1,4,10,13^. This hypothesis is supported by the fact that *acaA-*null mutant cells can aggregate and develop when prestimulated with uniform cAMP pulses^32^ or when PKA, which is downstream of the cAMP signaling pathway, is constitutively activated^19^. Monitoring [cAMP]_i_ levels using Flamindo2 showed that *acaA*^−^ cells could aggregate and form migrating slugs without [cAMP]_i_ oscillations after the cAMP pulse treatment (Fig. 5d, e). Previous reports^19,32^ and our investigation using mutants lacking cAMP relay imply a development capacity without periodic cAMP signals. However, our approach using wild-type cells shows for the first time that the disappearance of periodic cAMP signals occurs even with normal developmental. Therefore, we concluded that oscillatory cAMP signaling is not necessary for collective cell migration at the slug stage. This conclusion does not exclude the possibility that cAMP signals affect slug movement. The existence of optical density waves, which act as an index of cAMP relay in slugs, is controversial^12,13^. Therefore, it is possible that other experimental conditions would allow us to detect [cAMP]_i_ wave propagation using Flamindo2. Additionally, we found that [cAMP]_i_ and slug movement are sensitive to external cAMP stimuli (Fig. 4d), indicating that collective cell migration could depend on any endogenous cAMP relay that occurs in slugs.

The simultaneous monitoring of [cAMP]_i_ and cell sorting in *Dictyostelium* cells (Fig. 6a–c) suggested that the transition of cAMP signaling dynamics was a developmentally regulated event. This conclusion was confirmed in mutants that were developmentally arrested at the mound stage and showed no transition in cAMP signaling dynamics during development (Fig. 6d–f). In *Dictyostelium* cells, the expression pattern of genes essential for cAMP signaling is dramatically changed after slug formation^42^ (Supplementary Fig. 10). For example, the expression of high-affinity cAMP receptor cAR1 is high and seen in all cells during the aggregation stage, but becomes low after the mound stages^43,44^. In contrast, after slug formation, the expression of the high-affinity receptor cAR3 is seen in prespore cells, but the low-affinity receptors cAR2 and cAR4 are expressed in prestalk cells^45,46^. These expression changes are consistent with the sensitivity of the [cAMP]_i_ response to external cAMP stimulation at the multicellular phase being lower than at the unicellular phase (Fig. 4b and Supplementary Fig. 2c) and that the responsiveness of chemotaxis to cAMP gradients becomes weaker with mound formation^47^. The different sensitivities suggest changes in the cAMP signaling systems during multicellular formation. It has been reported that full-length CRAC is stably expressed under the control of a constitutively active promoter until the mound stage, but its expression is downregulated in slugs^29^. Therefore, our results suggest the active downregulation of molecules that mediate cAMP signaling and the developmental regulation of gene expression patterns during slug formation are involved in the transition of cAMP signaling dynamics. Additionally, one previous report showed that cAR1 is intrinsically internalized at the mound stage^48^, suggesting the possibility that the changes in protein localization and thus protein function also contributes to the transition of cAMP signaling dynamics.

**Fig. 7:**
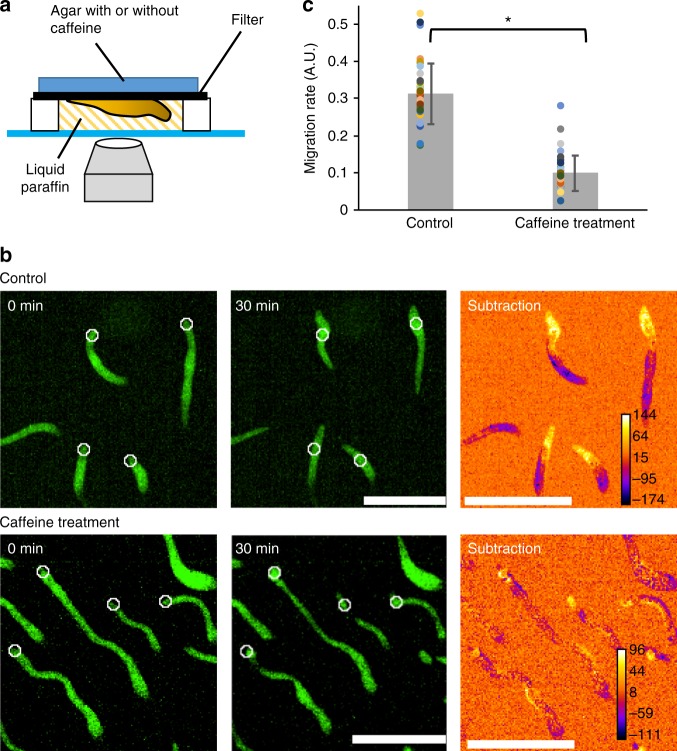
Caffeine treatment inhibits slug migration. **a** The scheme of experiments for monitoring the effect of caffeine on slug migration. Slugs were formed on a filter. Agar containing 0 mM (control) or 4 mM caffeine was put on the filter during the observation. **b** Snapshots of migrating slugs with or without 4 mM caffeine treatment. Fluorescent images of slugs expressing Citrine at *t* = 0 (left) and 30 min (middle) and subtracted images of the two (right) are shown. In the subtracted images, the yellow zones around the tips of the slugs indicate the migration space for 30 min. White circles shown in the *t* = 0 and 30 min images indicate the positions of the tips of the slugs at *t* = 0 min. Scale bars, 500 μm. **c** Comparison of migration rates between no treatment (Control) and caffeine-treated slugs (Caffeine treatment). *n* = 36 slugs for both groups (mean ± SD). Dots represent original data. *P < 10^–20^, Student’s two-tailed *t*-test

Our study revealed the disappearance of oscillatory cAMP signaling after multicellular slug formation and suggests the presence of mechanisms other than cAMP relay for the organization of collective cell migration in slugs. One possible mechanism is that extracellular cAMP signals such as a steady gradient and/or oscillations in slugs guide the direction of multiple cell movements. Blocking the cAMP signal pathway by caffeine treatment results in arresting the slug migration^18^, although the morphology of the slug is maintained under caffeine treatment by cell–cell adhesions and the extracellular matrix (Fig. 7 and Supplementary movie 10). Furthermore, although the developmental arrest phenotype of *acaA*^−^ cells can be rescued by the expression of constitutively active PKA^19^, mutant cells lacking *acaA* and *acrA*, which encode ACA and ACB, respectively, cannot form normal multicellular bodies even if constitutively active PKA is expressed^49^. These findings indicate that cAMP is still required for the collective migration of slugs in spite of cAMP oscillations being absent in migrating slugs. In order to clarify the role of cAMP in slugs, molecular genetics approaches and more sensitive cAMP measurements are required. Additionally, it is possible that other chemoattractants such as pterin^50^ and anterior-posterior Ca^2+^ gradients in slugs^51^ are also involved in the organization of collective cell migration in slugs.

In addition to chemical cues, it is widely known that collective migration is regulated by physical guidance cues through cell–cell contacts in higher organisms during events such as epithelial wound healing and closure^3,52^. Numerical simulations have shown that physical interactions between cells can organize collective cell migration in the absence of any external chemical signals^53,54^. These studies suggest the possibility that the collective cell migration in *Dictyostelium* multicellular bodies is organized by physical guidance cues rather than chemical guidance cues. One possible explanation is provided by “contact following”, which describes how cells follow other cells with which they have direct physical contact^55^. In fact, mutant cells which lack chemotaxis toward cAMP due to chemical mutagenesis show an organized collective migration that is mediated by cell–cell adhesions^56^. The adhesion proteins TgrB1/TgrC1 mediate cell alignment through head-to-tail cell contacts, leading to cell polarization including PIP3 localization and collective cell migration at later development stages^47^. The effects of adhesion proteins suggest that cell–cell contacts play important roles in multicellular morphogenesis.

Overall, we demonstrated directly the disappearance of cAMP signal oscillations and propagations between cells at the multicellular phase of *Dictyostelium*. Our work calls for reconsideration of the role cAMP relay has on collective cell migration in *Dictyostelium* and proposes a possibility that alternative mechanisms to cAMP relay contribute to the organization of collective cell migration at the multicellular phase.

## Methods

### Cell strains and culture conditions

The *Dictyostelium discoideum* cell strains used in this study are as follows: Ax2 (wild type), *acaA*^−^^57^, *gc*^−^ (*gca*^−^/*sgcA*^−^)^58^, and *gbfA*^−^. Ax2 cells were provided from the Dr. Guenther Gerisch laboratory (Max Planck Institute of Biochemistry) by Dr. Kei Inouye (Kyoto University). *acaA*^−^ cells were kindly provided by Dr. Satomi Matsuoka (RIKEN). *gc*^−^ cells were laboratory stocks. *gbfA*^−^ cells were obtained from the NBRP-nenkin Stock Center. Cells were grown axenically in HL5 medium (Formedium, UK) in culture dishes or shaking flasks at 21 °C. Transformants were maintained at 20 μg ml^−1^ G418 or 10 μg ml^−1^ BlastcidinS.

### Plasmid construction and genetic manipulation

The plasmids used in this study are as follows: pEcmAO-RFPmars, pHistone2B-RFP, pBIG_PH_Akt/PKB_-GFP^59^, pHK12neo_Citrine, and pHK12neo_Flamindo2. pBIG_PH_Akt/PKB_-GFP is from a laboratory stock. pEcmAO-RFPmars plasmid was obtained from the dictyBase Stock Center. pHistone2B-RFP plasmid was kindly provided by Dr. Tetsuya Muramoto (Toho University). pHK12neo_Citrine was constructed by the insertion of Citrine fragments into the *Bgl*II and *SpeI* sites of pHK12neo by the In-Fusion technique (Clontech laboratories Inc.). pHK12neo_Flamindo2 was constructed by the insertion of synthesized Flamindo2 fragments (GenScript) into the *Bgl*II and *Spe*I sites of pHK12neo. The codon usages of the Flamindo2 sequence were optimized to those of *D*. *discoideum* for efficient protein expression in *Dictyostelium* cells. Vectors except for pEcmAO-RFPmars allow constitutive expression of the proteins in cells under the *act15* promoter. The wild-type strain and mutant cells were transformed with ~1.5 μg plasmid by electroporation^60^, and transformants were selected with G418 and BlastcidinS at a final concentration of 20 μg ml^−1^ and 10 μg ml^−1^, respectively.

### Immunoblot analysis

Cells were developed on a cellulose membrane filter (see the Method subsection titled ‘Verification of proper function of Flamindo2 as the cAMP sensor at the slug stage’) and harvested at 5 and 12 h after starvation. Vegetative and developed cells were lysed by 4 × SDS sample buffer (Wako Pure Chemical Industries, Ltd.) and boiled at 95 °C for 5 min. Proteins from 4 × 10^5^ cells were blotted onto a polyvinylidene difluoride membrane and reacted with a polyclonal anti-GFP antibody (Anti-GFP pAb-HRP-DirecT, Code No. 598–7, Medical & Biological Laboratories) diluted 1:1000 in blocking solution (1% skim milk in TBS-T). Signals were visualized by the chemiluminescence of reactions with HRP substrate (Luminata^TM^ Forte Western HRP Substrate, Millipore), and images were acquired with ChemiDoc^TM^ XRS (BioRad).

### Instruments for image acquisition and analysis

In all experiments, cells were observed at 22 °C. Confocal images including a series of Z-stacks were taken by a confocal laser microscope (A1 confocal laser microscope system, Nikon) with an objective (Plan Apo VC 20 × /0.75 NA, Nikon) and oil immersion lenses (Plan Fluor 40×/1.30 NA and Apo TIRF 60×/1.49 NA, Nikon) or an inverted microscope (Eclipse Ti, Nikon) equipped with a CSU-W1 confocal scanner unit (Yokogawa), two sCMOS cameras (ORCA-Flash4.0v3, Hamamatsu Photonics) and objective lenses (Plan Fluor 4 × /0.13 NA, Plan Apo 10 × /0.45 NA and Plan Apo 20 × /0.75 NA, Nikon). Flamindo2, GFP and Citrine were excited by a 488 nm solid-state CW laser, and mRFPmars was observed using a 561 nm solid-state laser. Epifluorescence imaging was taken by using an inverted epifluorescence microscope (IX83, Olympus) equipped with a 130 W mercury lamp system (U-HGLGPS, Olympus), sCMOS cameras (Zyla4.2, Andor Technology or Prime 95B, Photometrics) and objective lenses (UPLSAPO 4×/0.16 NA and UPLSAPO 20×/0.75 NA, Olympus). Flamindo2 and tetramemethylrhodamine-maleimide (TMR, Invitrogen) were observed using fluorescence mirror units U-FGFP (Excitation BP 460–480, Emission BP 495–540, Olympus) and U-FMCHE (Excitation BP 565–585, Emission BP 600–690, Olympus), respectively. All images were processed and analyzed by Fiji and R software. For cell tracking, laboratory-made software was used^61^. In the sequential time-lapse images, the positions of each cells at the first frame were set manually from the fluorescence of Histone-RFP and movements of each cells were tracked automatically. Changes in Flamindo2 signals at the individual cell level were estimated by measuring the mean intensities of Flamindo2 in the regions (~3–5 μm^2^ regions) positioned on the cytosol of the tracked cells at each time point. Cell velocity was calculated by dividing the displacement between two sequential frames by the interval time, and the unit of velocity was converted to μm per min. The period of an oscillation was calculated by averaging the difference between the peaks of the oscillation (Flamindo2 signals and cell velocity data) or defined as the first largest peak at *t* > 0 of the autocorrelation function of the PH_Akt/PKB_-GFP translocation data. Data with at least three peaks in the oscillation were used for the analysis. In general, the fluorescence intensities of Flamindo2 were normalized with values at *t* = 0.

### Image acquisition of *Dictyostelium* development

To induce starvation and development, cells were harvested during the exponential phase (1.5–3 × 10^6^ cells ml^−1^) and washed three times in KK2 phosphate buffer (20 mM KH_2_PO_4_/K_2_HPO_4_, pH 6.0). In this study, two methods were used to observe development on agar. To observe early aggregation, cells were plated on the entire surface of 2% water agar plates (2% w/v Difco Bacto-agar in ultrapure water) at a density of 5–7 × 10^5^ cells cm^−2^ and incubated at 21 °C. To observe the mound and slug stages, 5 μl of cell suspension at a density of 2–4 × 10^7^ cells ml^−1^ was deposited on 1.5% water agar and incubated at 21 °C for 6–15 h. To image development, a method described previously^62^ was used. Here, a piece of agar was cut out and placed upside down on a 35-mm glass bottom dish (12 mm diameter glass, Iwaki) directly or on a spacer (thickness: 50, 100, or 150 μm) attached to the dish. The spacer was filled with liquid paraffin (Nacalai Tesque) to avoid light scattering. To prevent desiccation during the observation, wet paper was placed in the dish and the agar piece was covered with liquid paraffin. In this condition, cells and multicellular bodies could move freely and develop normally for more than 12 h under the microscope. The Z series of fluorescence images was taken by the confocal microscope at 10–30 s intervals.

In addition to the above methods, we also applied the technique “2D slug”^63,64^ for efficient cell tracking in slugs because three-dimensional (3D) scroll movement of the slug and thickness of the tissue make it difficult to follow individual cell movements in normal slugs. One microlitre of cell suspension at a density of 4 × 10^7^ cells ml^−1^ was deposited on 2% water agar plates together with 2 μl liquid paraffin. A coverslip was placed over the suspension, which was incubated at 22 °C for more than 15 h. The Z series of the fluorescent images was acquired at 15-s intervals for 20–30 min by the confocal microscope. The 2D slug, which has only few (~4) cell layers and thus enables us to follow cell movement easily, showed similar properties with normal slugs with respect to cell movement and proportion of cell types^63,64^. We observed periodic cell movement with no obvious [cAMP]_i_ oscillation in both normal slugs and 2D slugs (Fig. 3e and Supplementary Fig. 6c, d).

### Verification Flamindo2 as an indicator of [cAMP]_i_ changes in *Dictyotelium* cells at the unicellular phase

Cells expressing Flamindo2 or Citrine were starved in 1 ml of developmental buffer (DB: 5 mM Na/KPO_4_, 2 mM MgSO_4_, 0.2 mM CaCl_2_, pH 6.5) at a density of 5 × 10^5^ cells ml^−1^ for 1 h and incubated for a subsequent 5 h in the presence of 100 nM cAMP pulses given at 6-min intervals. Cells were then washed three times with 1 ml DB and resuspended in DB at a density of 10^6^ cells ml^−1^. Forty microlitre of the cell suspension was dropped onto a glass bottom dish. Cells were stimulated by adding 160 μl cAMP solution (the target concentration of cAMP) to the cell droplet. In caffeine experiments, cells were exposed to 4 mM caffeine for 30 min on a glass bottom dish before cAMP stimulation. Fluorescent images were acquired by the confocal microscope at 5-s intervals during stimulation. Averaged fluorescence intensities of Flamindo2 or Citrine in 5 μm^2^ regions positioned within the cytosol were measured at each time point.

To confirm that Flamindo2, which has an EC_50_ of 3.2 μM to cAMP and Hill coefficient of 0.95^22^, can cover the range of cytosolic cAMP levels in *Dictyostelium* cells, we estimated intracellular cAMP concentrations based on three parameters: cAMP concentration (about 2–75 pmol mg^−1^ protein^8,16,37^), protein amount per cell (7 × 10^–8^ mg cell^−1^: calculated based on the notation that 10^9^ cells equals about 1 g wet cells, which equals ~70 mg protein^39^) and cell volume (0.43 pl cell^−1^ ^38^). For example,

2 (pmol mg^−1^) × 7 × 10^–8^ (mg cell^−1^)/0.43 (pl cell^−1^) = 0.32 μM

Our calculation showed that the intracellular cAMP concentration in unstimulated and cAMP-stimulated *Dictyostelium* cells at all development stages is ~0.3–12 μM.

### Verification of proper function of Flamindo2 as the cAMP sensor at the slug stage

Cells expressing Flamindo2 and *ecmAO*::mRFPmars were washed and deposited on a cellulose membrane filter (Advantec) at a density of 5 × 10^5^ cells cm^−2^ and incubated at 21 °C for 12 h to allow slug formation. The slugs were harvested in DB and dissociated into single cells by repeated passages through a 25 G needle (Terumo) with a 1 ml syringe on ice^65^. Slug-disaggregated cells were resuspended in DB at a density of 10^6^ cells ml^−1^, and 40 μl of the suspension was deposited onto a 12-mm glass bottom dish. cAMP stimulation and caffeine treatment were performed as described in the Method subsection titled ‘Verification Flamindo2 as an indicator of [cAMP]_i_ changes in *Dictyotelium* cells at the unicellular phase’. Fluorescent images were acquired by the confocal microscope at 5-s intervals during stimulation. Averaged fluorescence intensities of Flamindo2 in 5 μm^2^ regions positioned within the cytosol were measured at each time point. Cell types of slug-disaggregated cells were distinguished by the intensity of *ecmAO*::mRFPmars.

We also performed the cAMP stimulation test on intact slugs by cAMP microinjection into water agar (Fig. 4c). Cells were developed on 2% water agar plates until slug formation, and a piece of agar was cut out and placed directly on a glass bottom dish. To avoid light scattering and desiccation, the agar piece was covered with liquid paraffin. A Femtotip microcapillary (1 µm tip diameter, Eppendorf) filled with 10 mM cAMP diluted in ultrapure water was mounted onto a Femtojet pump and micromanipulator (Eppendorf). To visualize the diffusion of cAMP in the water agar after injection, 10 μM TMR was added to the cAMP solution. The injection pressure and injection time were set to 1500 hPa and 0.1 s, respectively. In this condition, 20 pl of solution was emitted from the microcapillary. The volume of injected solution was calculated from the diameter of spherical droplets of the cAMP solution injected in a drop of liquid paraffin. A tip of the capillary was touched to the agar surface near a slug, and cAMP solution was injected into the agar. The stimulation was applied at 6-min intervals as described previously^13^. Fluorescent and DIC images were acquired at 15-s intervals by the IX83 epifluorescence microscope.

### Investigation of PH_AKT/PKB_-GFP translocation to cAMP-stimulated cells dissociated from multicellular bodies or intact slugs

Cells expressing PH_AKT/PKB_-GFP were washed and deposited on a cellulose membrane filter at a density of 5 × 10^5^ cells cm^−2^ and incubated at 21 °C for 12 h to allow slug formation, or 5 μl of cell suspension at a density of 4 × 10^7^ cells ml^−1^ was deposited on 2% water agar and incubated at 21 °C for 6 h to allow loose mound formation. Mechanical dissociation of cells from loose mounds or slugs was performed as described in the Method subsection titled ‘Verification of proper function of Flamindo2 as the cAMP sensor at the slug stage’. Forty microlitre of the cell suspension (loose mound cells, 5 × 10^5^ cells ml^−1^; slug cells, 2 × 10^6^ cells ml^−1^) was deposited onto a 12-mm glass bottom dish and allowed to settle for 5 min (loose mound cells) or 20 min (slug cells). Cells were stimulated by 10 μM cAMP as described in the Method subsection titled ‘Verification Flamindo2 as an indicator of [cAMP]_i_ changes in *Dictyotelium* cells at the unicellular phase’. Fluorescent images were acquired by the confocal microscope at 5-s intervals during stimulation. Averaged fluorescence intensities of PH_AKT/PKB_-GFP in 4 μm^2^ regions positioned within the cytosol were measured at each time point.

In addition, we performed the cAMP stimulation test on intact slugs expressing PH_AKT/PKB_-GFP by cAMP microinjection into water agar as shown in Fig. 4c. To acquire fluorescent images of PH_AKT/PKB_-GFP in slugs with high magnification, slugs were formed on thin agar covered directly on the glass bottom dish. Here, 100 μl of melting agar was poured into a well of 12-mm glass bottom dish and then 65 μl of agar was sucked up, resulting in the formation of a uniform thin agar layer on the glass. Five microlire of cell suspension at a density of 4 × 10^7^ cells ml^−1^ was deposited at the center of the thin agar layer and incubated at 21 °C for 12 h to allow for slug formation. The manipulation of cAMP microinjection was performed as described in the Method subsection titled ‘Verification of proper function of Flamindo2 as the cAMP sensor at the slug stage’.

### Induction of *acaA-*null cell aggregation and slug formation by exogenous cAMP pulses

The method by Pitt et al.^32^ was applied with some modification. *acaA*-null cells expressing Flamindo2 were washed and suspended in 1 ml DB at a density of 6 × 10^6^ cells ml^−1^. The cells were then incubated on a 35-mm plastic dish (Iwaki) for 4 h and stimulated with 30 nM cAMP pulses at 6-min intervals. Cells were then washed and suspended in 1 ml DB again and subsequently incubated on a 35-mm plastic dish or a 35-mm glass bottom dish (with 27-mm diameter glass, Iwaki) for more than 12 h with 30 μM cAMP pulses at 60-min intervals. After terminating the exogenous cAMP pulse treatment to induce multicellular formation, *acaA*-null cells were washed and resuspended in DB at a density of about 4 × 10^7^ cells ml^−1^, and 5 μl of cell suspension was deposited on 2% water agar plates because a submerged culture inhibits the multicellular development of even wild-type cells. Time-lapse images during the development were acquired at 30-s intervals by the confocal microscope. We confirmed that the clumps of *acaA*-null cells could not synthesize cAMP in response to external cAMP signals by monitoring Flamindo2 signals (Fig. 5c). After the cAMP pulses, the cell clumps were washed by DB three times and then resuspended in 450 μl DB on a glass bottom dish. Fifty microlitre of 1 mM cAMP (final concentration: 100 μM) was applied to the dish under observation of the microscope. Fluorescent images were acquired by the confocal microscope at 5-s intervals during the stimulation.

### Monitoring the effect of caffeine treatment on slug migration

Cells expressing Citrine were washed, and 5 μl of cell suspension at a density of 4 × 10^7^ cells ml^−1^ was deposited on a cellulose membrane filter and incubated at 21 °C for 12 h to allow for slug formation. A piece of filter was cut out and placed upside down on a spacer (thickness, 100 μm) attached to a glass bottom dish. The spacer was filled with liquid paraffin to avoid light scattering. A piece of 2% water agar with or without 4 mM caffeine was then put on the filter (Supplementary Fig. 11a). In the caffeine treatment experiments, the dishes were settled for 5 min before observation to allow the caffeine to permeate through the filter. Time-lapse images of slug migration were acquired at 30-s intervals for 30 min by the confocal microscope. Migration was calculated by measuring the displacement between the positions of the slug tip at 0 and 30 min. Because the speed of slug migration varies depending on the slug size^66^, the migration distance for 30 min divided by each slug length was regarded as the migration rate.

## Supplementary information

Supplementary Information

Description of Additional Supplementary Files

Supplementary Movie 1

Supplementary Movie 2

Supplementary Movie 3

Supplementary Movie 4

Supplementary Movie 5

Supplementary Movie 6

Supplementary Movie 7

Supplementary Movie 8

Supplementary Movie 9

Supplementary Movie 10

Supplementary Data 1

Supplementary Data 2

Supplementary Data 3

Supplementary Data 4

Supplementary Data 5

Supplementary Data 6

## Data Availability

All data are presented in the manuscript or the supplementary materials. The source data underlying the graphs shown in the main figures are presented in the Supplementary Data 1–6. Other data supporting the findings of this study are available from the corresponding authors upon request. The plasmids generated in this study will be available at Dicty stock center (http://dictybase.org/StockCenter/StockCenter.html) and NBRP-nenkin (https://nenkin.nbrp.jp/locale/change?lang = en).
