# Supplementary material for: Collective cell migration of Dictyostelium without cAMP oscillations at multicellular stages
Source: Commun Biol. 2019 Jan 24;2:34. doi: 10.1038/s42003-018-0273-6 (PMC6345914; doi:10.1038/s42003-018-0273-6)
Supplement: Supplementary file 1 — Supplementary Information [file 42003_2018_273_MOESM1_ESM.pdf]

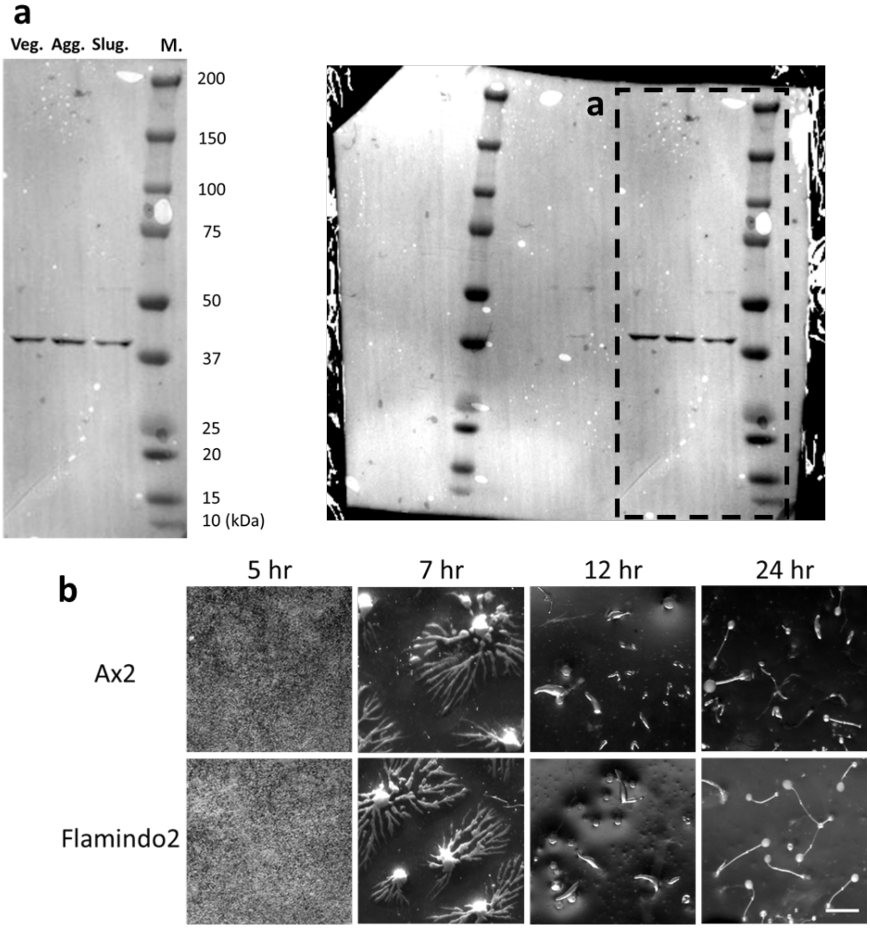

Supplementary Figure 1. Expression of Flamindo2 was stable throughout development and had no toxic effect on the development process. (a) Immunoblot analysis of lysates of cells expressing Flamindo2 at each developmental stage using a polyclonal anti-GFP antibody. Cells were developed on a filter and harvested 5 hr (early aggregation stage) and 12 hr (slug stage) after starvation. Experiments were performed 7 times and the representative blotting data of is shown here. Left, a cropped image. Right, original image of the immunoblot. Veg, vegetative stage (development, 0 hr). Agg, early aggregation stage. Slug, slug stage. M, molecular weight marker. (b) Developmental time course of cells expressing Flamindo2. Panels show developmental stages of Ax2 cells (upper panels) and cells expressing Flamindo2 (lower panels) on agar. Early aggregation (5 hr), aggregation stream (7 hr), tipped mounds and slugs (12 hr), and fruiting bodies (24 hr). Scale bar, 1 mm.

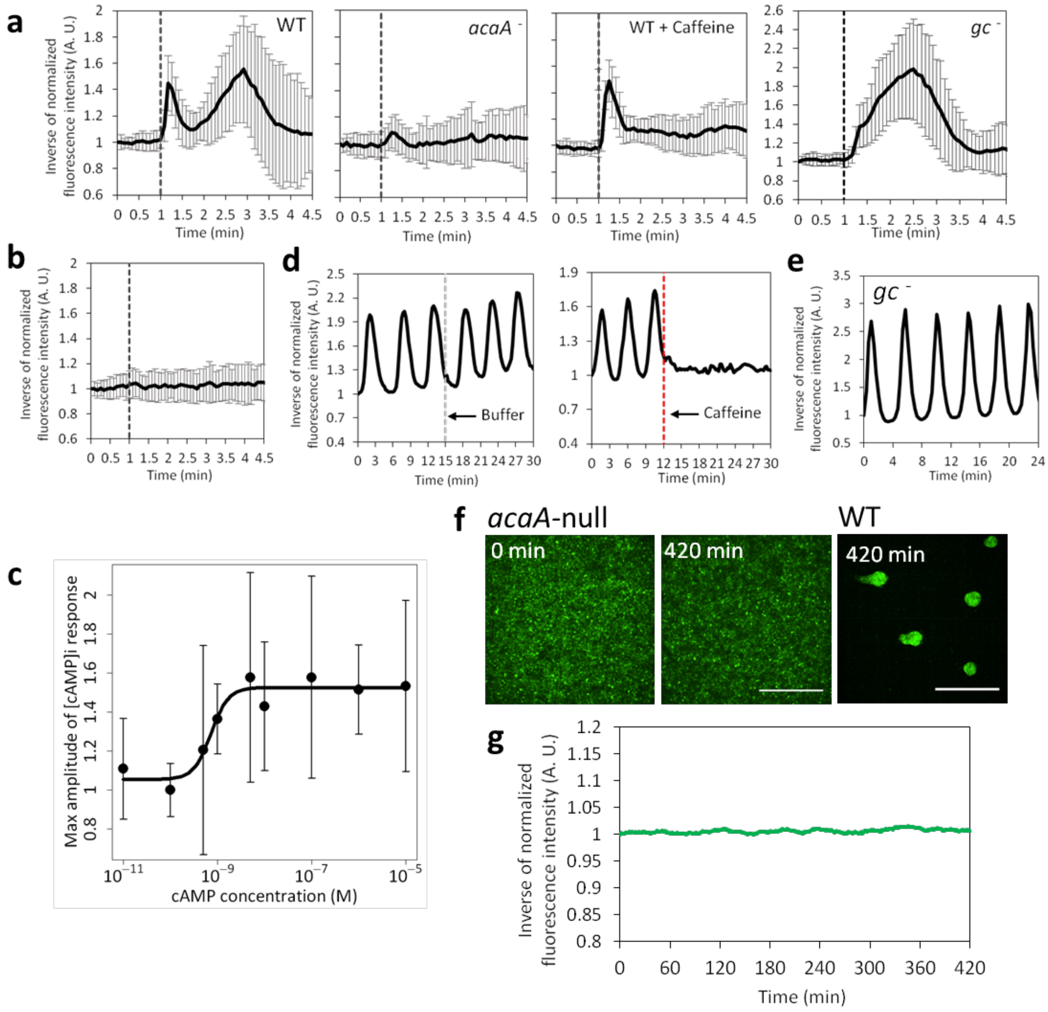

Supplementary Figure 2. Flamindo2 could detect changes in $[cAMP]_i$ in aggregation-competent cells. (a) Time-course plots of Flamindo2 signals in *Dictyostelium* Ax2 cells after 10 µM cAMP stimulation. The inverse of the fluorescence intensity of Flamindo2 is plotted on the y-axis (mean ± SD: n = 30 cells in all panels). First panel, wild-type (Ax2) cells; second panel, *acaA*-null cells; third panel, wild-type cells treated with 4 mM caffeine; fourth panel, gc^- cells. Dashed lines indicate the time point of the stimulation. (b) Time-course plot of the fluorescence intensity of Citrine in Ax2 cells (expressing only Citrine and not Flamindo2) after 10 µM cAMP stimulation (mean ± SD: n = 30 cells). (c) The dose-dependent curve of the $[cAMP]_i$ response to various cAMP stimulations (0.01 nM to 10 µM). The maximum amplitudes of the response were plotted against the cAMP concentration (mean ± SD). Sample number: 10 µM, n =30 cells; 10 nM, n = 19 cells; other data points, n = 20 cells. (d) Caffeine treatment inhibited the synchronous oscillations in the Flamindo2 signal. Cells were starved in developmental buffer on a glass bottom dish. Development buffer with or

without 4 mM caffeine (final concentration) was added to aggregating cell populations at the time points indicated by the dashed lines. The mean fluorescence intensity of Flamindo2 was measured in cell populations (318 μm^2 region), and the inverse of the intensity is plotted on the y-axis. (e) *gc*$^-$ cell populations also showed synchronous oscillations in the Flamindo2 signal during aggregation. *gc*$^-$ cells were starved in developmental buffer on a glass bottom dish. The mean fluorescence intensity of Flamindo2 was measured in aggregating cell populations (250 μm^2 region), and the inverse of the intensity is plotted on the y-axis. (f) Defective aggregation in mutant cells lacking *acaA*. Fluorescent images of *acaA*-null and wild-type cells expressing Flamindo2 after starvation are shown. Wild-type cells formed mounds, while *acaA*-null cells did not aggregate after 7 hours of starvation. Scale bars, 500 µm. (g) Time course plot of Flamindo2 signals in *acaA*-null cells after starvation. The fluorescence intensity of Flamindo2 in a 100 μm^2 region in the left panels of (f) was measured, and the inverse of the fluorescence intensity is plotted against time.

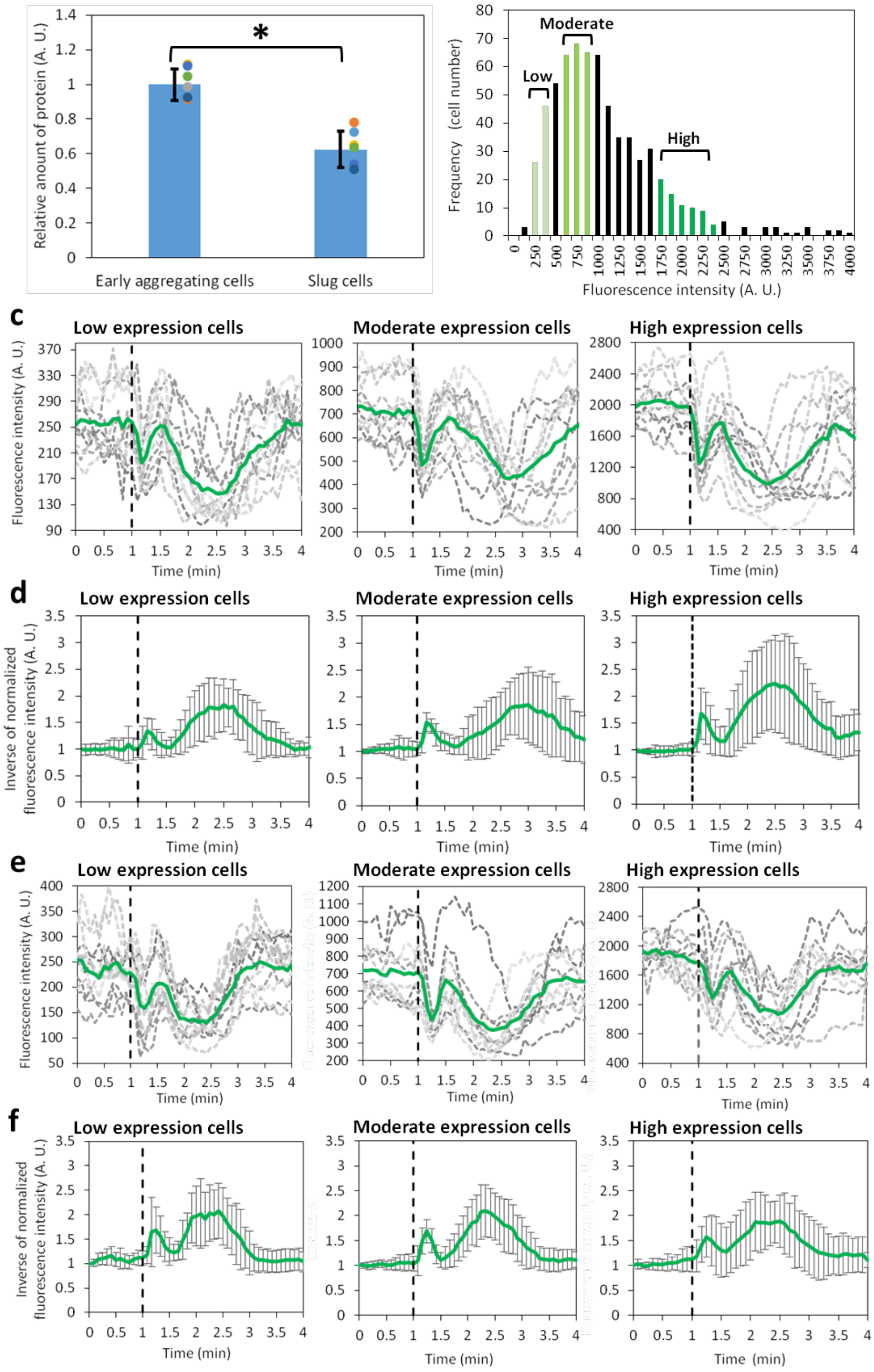

Supplementary Figure 3. Sensitivity of Flamindo2 signal response to external cAMP stimulation was independent of Flamindo2 expression level. (a) Comparison of expression levels of Flamindo2 between early aggregating cells (5 hr starvation) and slug cells. Chemiluminescent signals of Flamindo2 from immunoblot analyses were quantified and normalized (mean ± SD: n = 7 samples). Dots plotted on the graph are original data.

*: $P < 10^{-7}$, Student's two-tailed *t*-test. (b) Histogram of the fluorescence intensities of Flamindo2 in chemotactic-competent cells (n = 657 cells). Mean intensity of Flamindo2 in 5 μm^2 regions positioned in the cytosol of cells starved for 5 hr was measured. The graph indicates the variation of the expression level of Flamindo2 in the cell populations. Based on the intensities, cells were classified into three representative groups: cells expressing Flamindo2 at low levels (125–375 A.U., light green), cells expressing Flamindo2 at moderate levels (500–1000 A.U., green), and cells expressing Flamindo2 at high levels (1625–2375 A.U., dark green). (c), (e) Time-course plots of Flamindo2 signals in cells of the three clades after (c) 10 µM or (e) 10 nM cAMP stimulation. The fluorescence intensity of Flamindo2 in 5 μm^2 regions positioned in the cytosol of cells was measured and plotted on the y-axis. In each graph, gray dashed lines show the data of 11 cells, and green lines show the average across those cells. (d), (f) Time-course plots of normalized Flamindo2 signals in cells of the three clades shown in (c) and (e), respectively. The fluorescence intensity of each cell shown in (c) and (e) was normalized at t = 0, and the inverse was averaged across the cells (mean ± SD). In (c) – (f), dashed black lines indicate the time points of the stimulation.

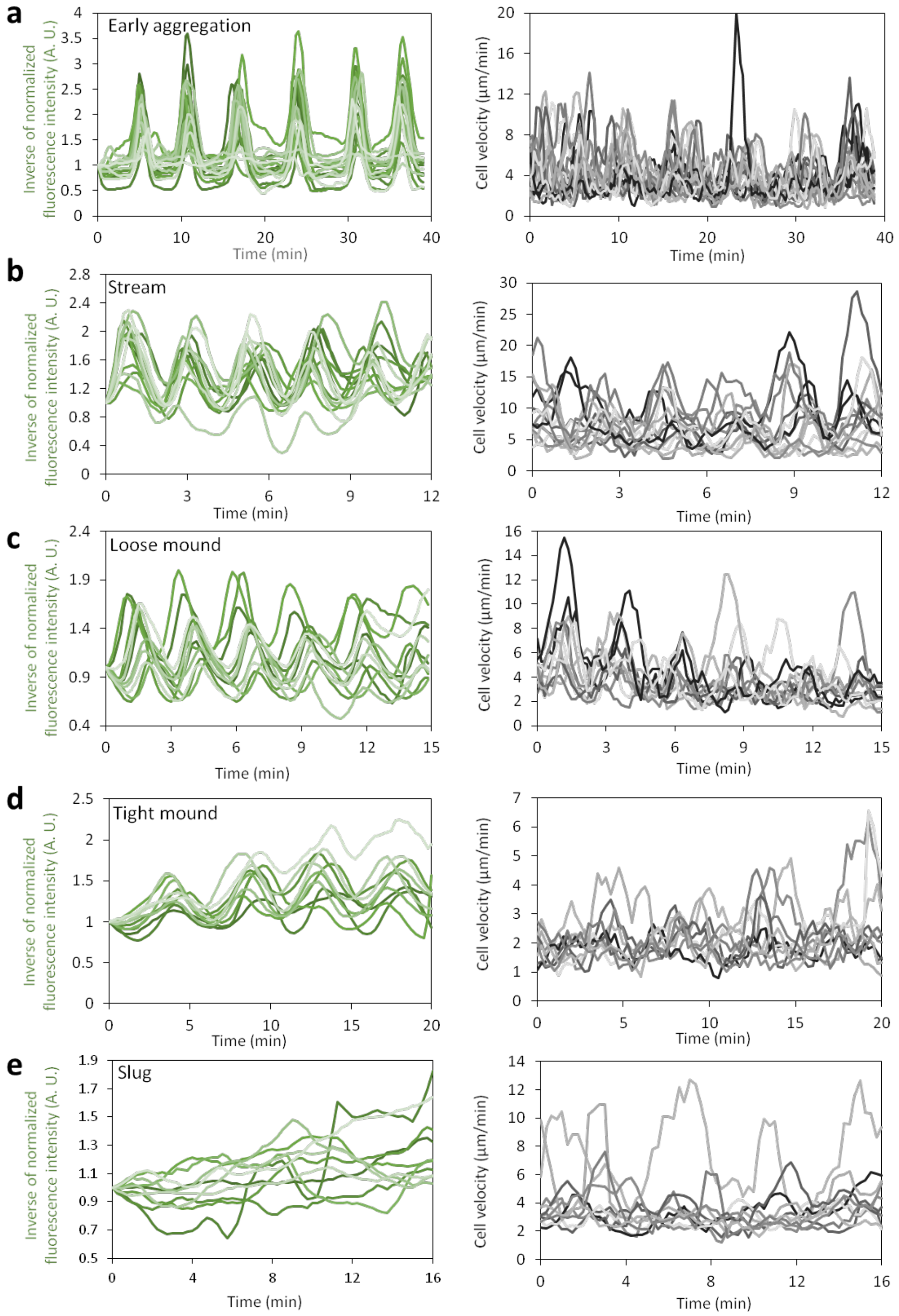

Supplementary Figure 4. Simultaneous monitoring of [cAMP]$_i$ and cell velocity in individual cells. Data of individual cells as the basis of the average data shown in Figure 3 are shown. Left graphs show Flamindo2 signals, and right graphs show cell velocities. The curves were smoothed by a running average over four data points. (a) Early aggregation (n = 20 cells). (b) Aggregation stream (n = 14 cells). (c) Loose mound (n = 12 cells). (d) Tight mound (n = 10 cells). (e) Slug (n = 10 cells).

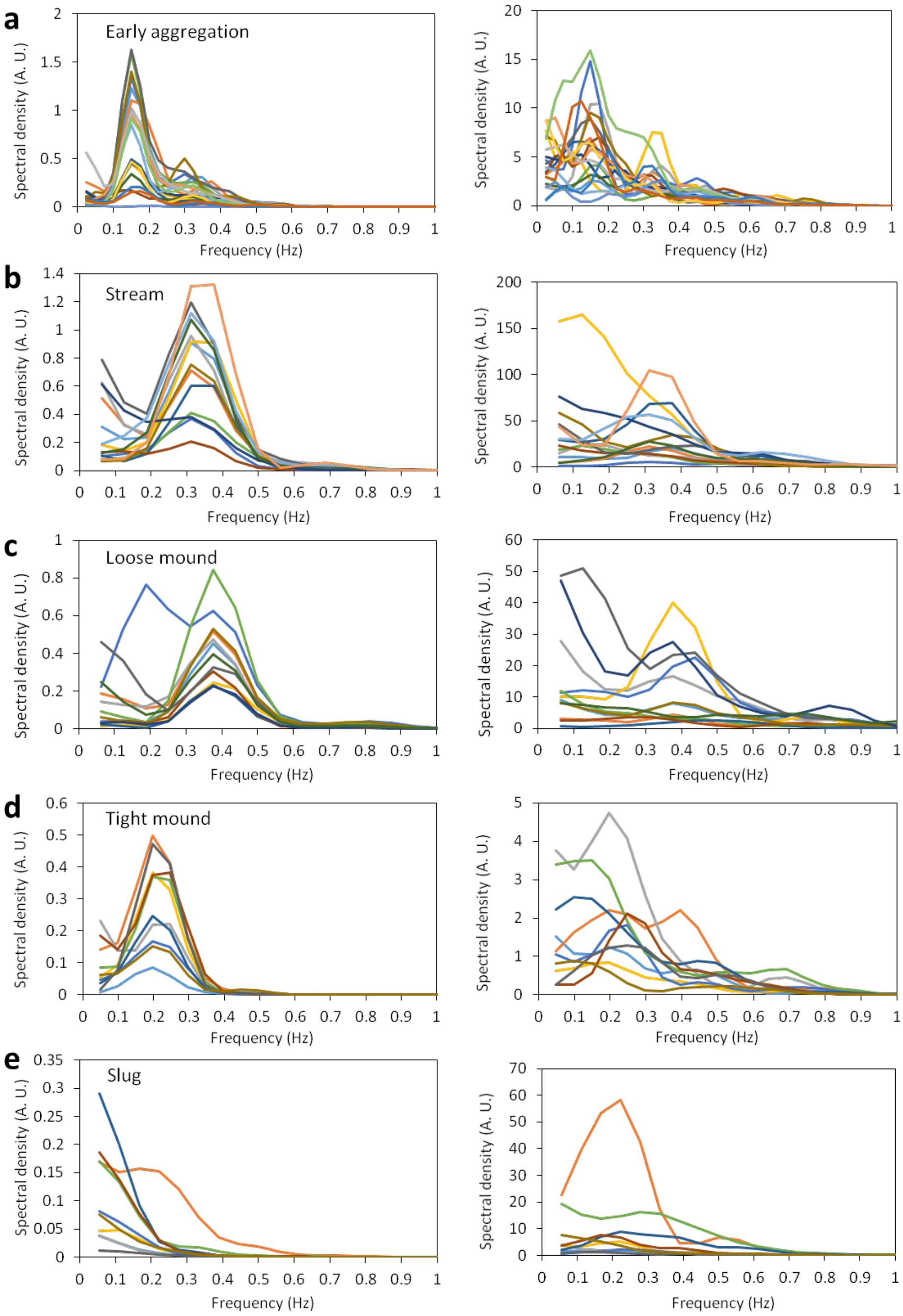

Supplementary Figure 5. Power spectrum of Flamindo2 signals and cell velocities of individual cells at each developmental stage. Power spectral analysis was performed on the data shown in Supplementary Figure 4. Spectral densities plotted against the frequency are shown. Left graphs show Flamindo2 signals, and right graphs show cell velocities. (a) Early aggregation (n = 20 cells). (b) Aggregation stream (n = 14 cells). (c) Loose mound (n = 12 cells). (d) Tight mound (n = 10 cells). (e) Slug (n = 10 cells).

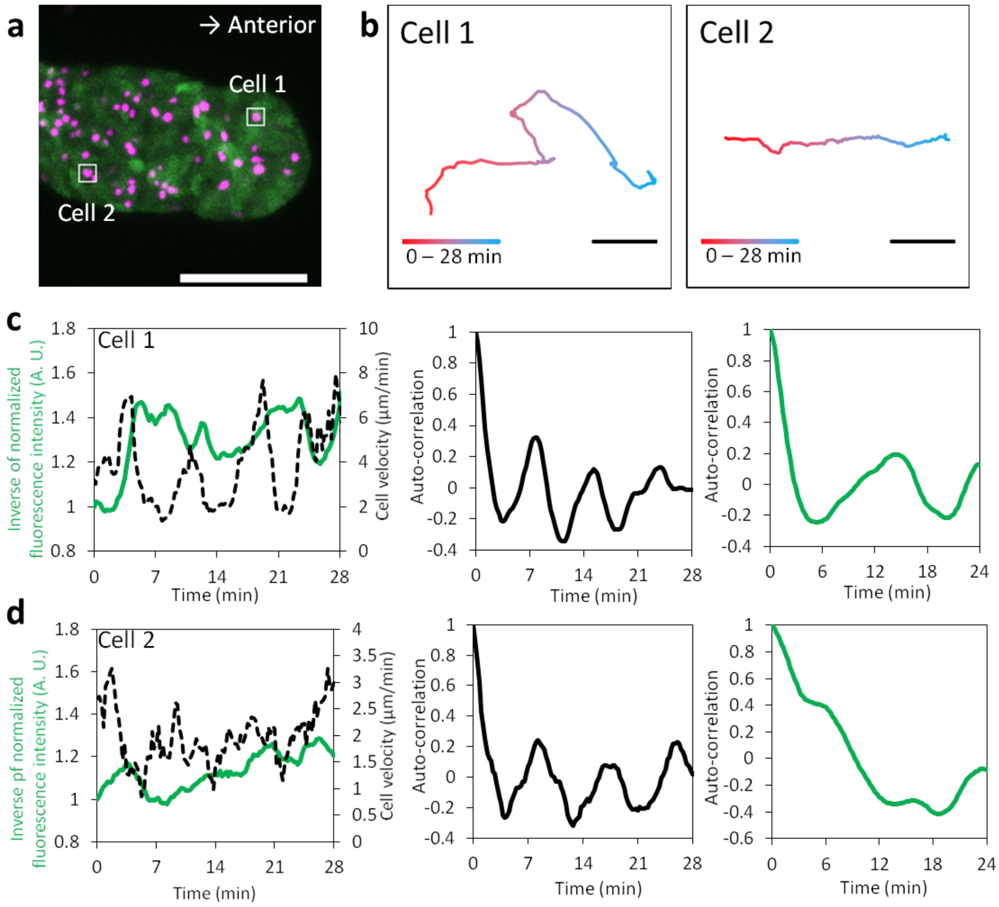

Supplementary Figure 6. [cAMP]ᵢ dynamics and cell movement in migrating slugs. (a) Fluorescent images of a slug expressing Flamindo2 mixed with 2% Histone2B-RFP-labelled cells. Scale bar, 50 µm. (b) Trajectories of prestalk (cell 1) and prespore (cell 2) cells for 28 minutes. Scale bars, 10 µm. (c), (d) Dynamics of Flamindo2 signals and movements of cell 1 and cell 2. Left graphs show the time-course plots of Flamindo2 signals (green solid line) and cell velocities (black dashed line). Middle and right graphs show the auto-correlation of the cell velocity and Flamindo2 signal, respectively.

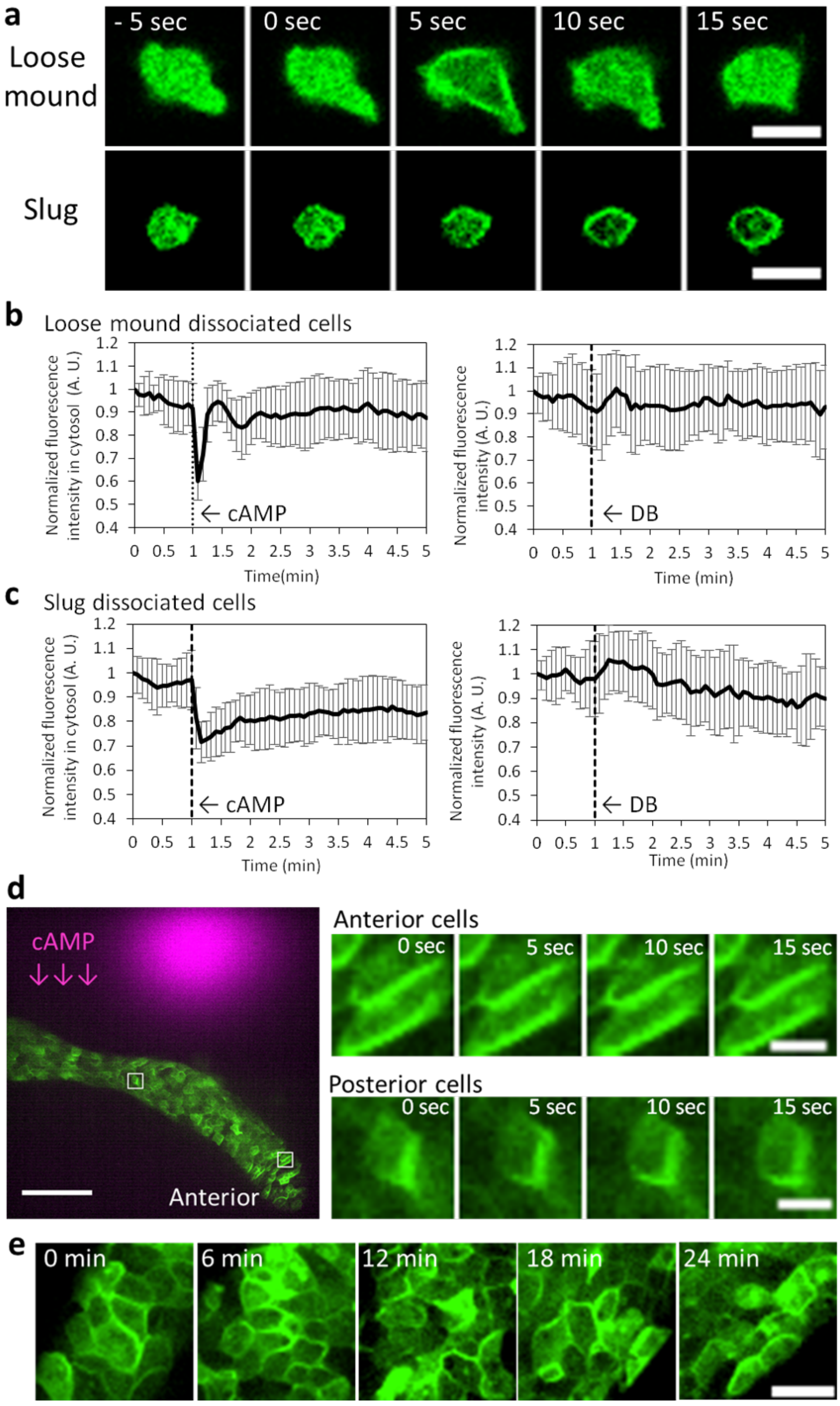

Supplementary Figure 7. Translocation of PH$_{AKT/PKB}$-GFP to the plasma membrane of cells from loose mounds or slugs in response to cAMP stimulation.

(a) Translocation of PH$_{AKT/PKB}$-GFP to the plasma membrane of cells dissociated from loose mounds (upper panels) or slugs (lower panels) in response to uniform cAMP stimulation. Scale bar, 5 μm. (b), (c) Time course plot of fluorescence intensity of

$PH_{AKT/PKB}$-GFP in the cytosol of cells dissociated from loose mounds or slugs (mean ± SD). Loose mound (n = 24 cells), slug (n = 30 cells). Dashed lines indicate the time point of the stimulation with developmental buffer (DB) with or without 10 μM cAMP. (d) External cAMP stimulation to an intact slug by injection of cAMP into agar near the slug from a microcapillary. Left, fluorescent image of $PH_{AKT/PKB}$-GFP in the slug and diffusing dye mixed with cAMP to visualize the injected solution during the stimulation. cAMP solution was diffused from the tip of the capillary positioned on the top of the image and stimulated the slug. Fluorescence images of the focal plane near the bottom of the slug were taken by a confocal microscope. Anterior part of the slug faces the bottom side of the image. Scale bar, 50 μm. Right, fluorescence images of $PH_{AKT/PKB}$-GFP in the anterior and posterior cells in the slug during the cAMP stimulation. The regions indicated by the white boxes in the left image were expanded and are shown in the right images. Time after the cAMP stimulation (seconds) is shown in the upper right of the images. Scale bar, 5 μm. (e) Interference of cAMP signaling in an intact slug by 4 mM caffeine. The caffeine treatment was performed as shown in Fig. 7a. Fluorescent images of $PH_{AKT/PKB}$-GFP in slug cells are shown. Anterior part of the slug faces the upper right side in each image. Time after the caffeine treatment (minutes) is shown in the upper left of the images. Scale bar, 10 μm.

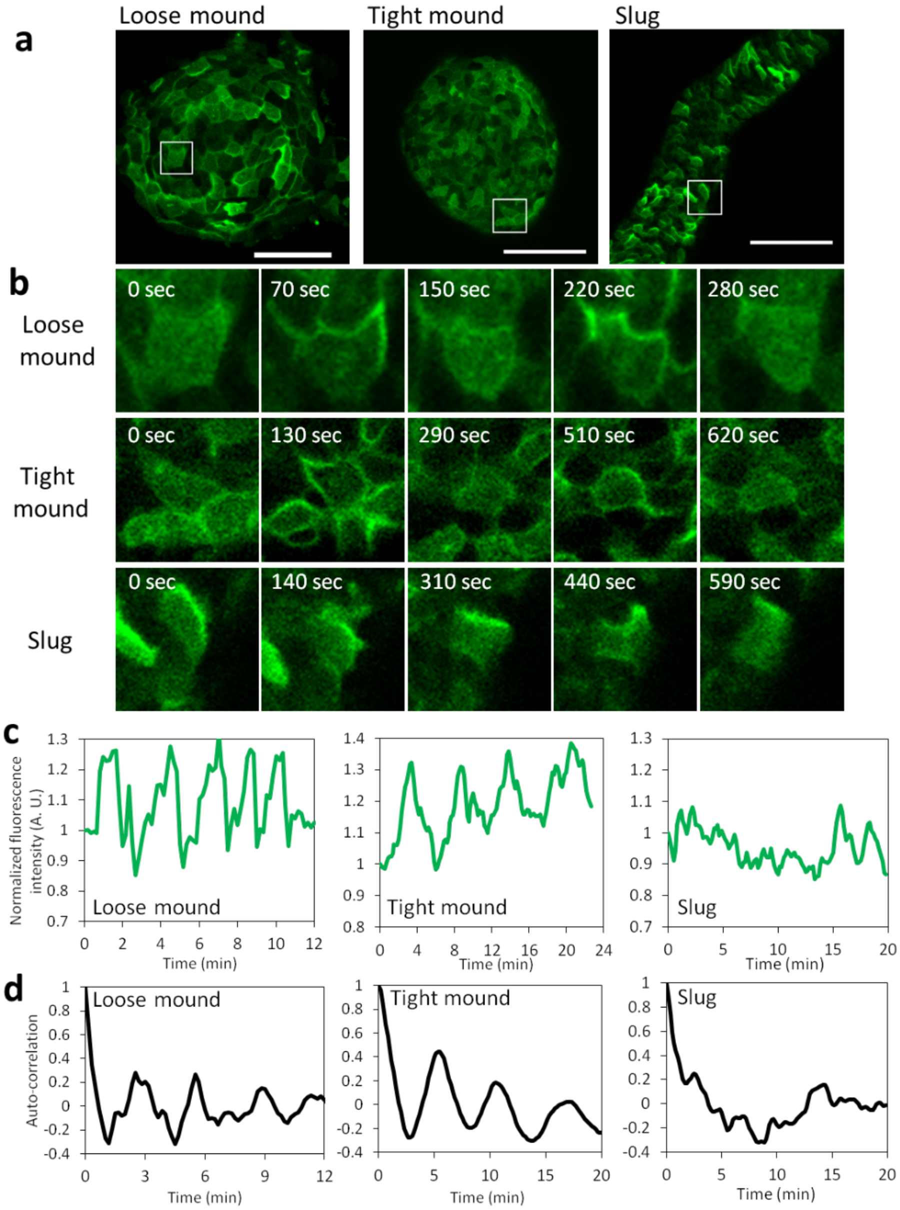

Supplementary Figure 8. Periodic translocation of $PH_{Akt/PKB}$-GFP to the leading edge of cells was observed in the mound stages, but not in the slug stage. (a) Fluorescent images of *Dictyostelium* Ax2 cells expressing $PH_{Akt/PKB}$-GFP in a loose mound, a tight mound and a slug. Scale bars, 50 µm. (b) $PH_{Akt/PKB}$-GFP localization to the leading edge of cells in the white boxes shown in (a). (c) Time-course plots of fluorescence intensity of $PH_{Akt/PKB}$-GFP in the cytosol. Mean intensity in a 3–5 μm^2 region positioned in the cytosol of a representative cell was measured. (d) Auto-correlation of the time course changes shown in (c).

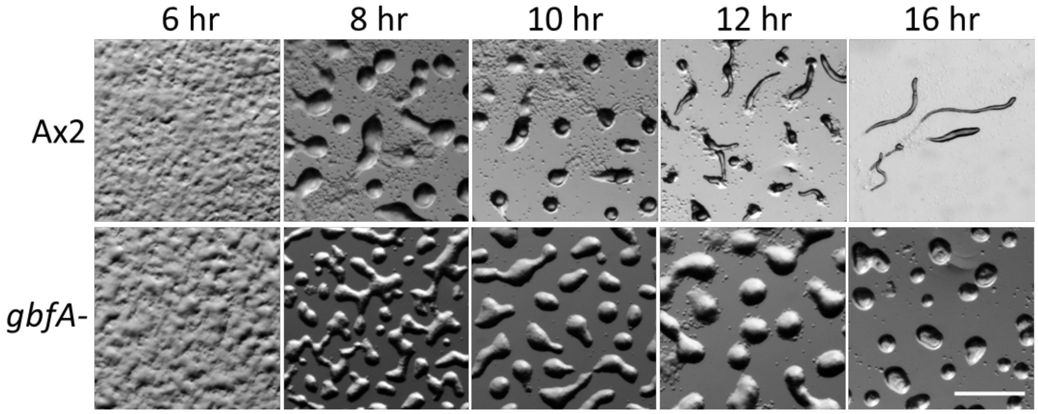

Supplementary Figure 9. Developmental time course of wild-type and *gbfA*$^-$ mutant cells. Cells were deposited on agar and allowed to develop. (Upper panels) Wild-type Ax2 cells show several developmental stages which could be distinguished by morphology: early aggregation (6 hr), loose mounds (8 hr), tight mounds (10 hr), and slugs (12, 16 hr). (Lower panels) Development of *gbfA*$^-$ mutant cells arrested at the loose mound stage (8 – 16 hr). Scale bar, 1 mm.

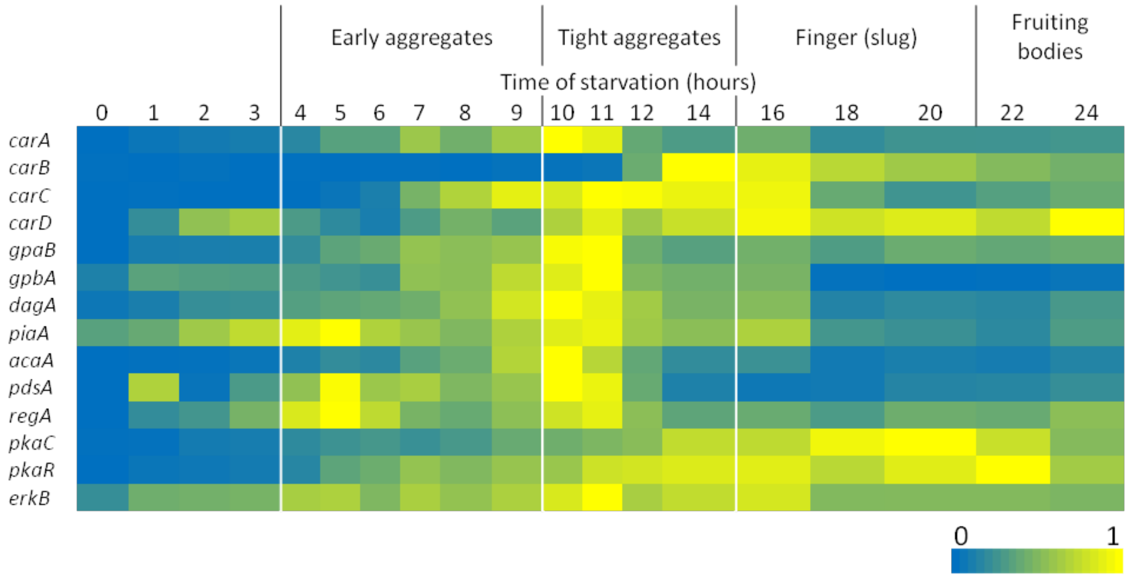

Supplementary Figure 10. Gene expression profile of genes involved in cAMP signaling along the time course of development. Gene expression profiles of cAMP receptors (*carA, carB, carC, carD*), G proteins (*gpaB, gpbA*), regulators (e. g., cytosolic regulator of adenylyl cyclase, *dagA*) and effectors (e. g., adenylyl cyclase, *acaA*) are shown. The profiles are based on RNA-seq data obtained from a web-based platform for the sequence data dictyExpress[1]. The indicated developmental time courses of the relative gene expression levels are based on the notation in Rosengarten *et al*[2].

Supplementary References

1. Stajdohar, et al. dictyExpress: a web-based platform for sequence data management and analytics in *Dictyostelium* and beyond. *BMC Bioinfomatics.* **18**, 291 (2017)
2. Rosengarten, et al. Leaps and lulls in the developmental transcriptome of *Dictyostelium discoideum*. *BMC genomics.* **16**, 294 (2015)
