# Supplementary material for: Collective cell migration of Dictyostelium without cAMP oscillations at multicellular stages
Source: Commun Biol. 2019 Jan 24;2:34. doi: 10.1038/s42003-018-0273-6 (PMC6345914; doi:10.1038/s42003-018-0273-6)
Supplement: Supplementary file 2 — Description of Additional Supplementary Files [file 42003_2018_273_MOESM2_ESM.pdf]

Description of Supplementary Movies

File name: Supplementary Movie 1
Description: Onset of $[cAMP]_i$ wave oscillations and wave propagations in cell populations during early aggregation and stream formation (see Fig. 1a for corresponding snapshots). Fluorescent images of Flamindo2 were acquired by an epifluorescence microscope at 20-second intervals. Data were obtained 3–7.5 hours after starvation. Scale bar, 1 mm.

File name: Supplementary Movie 2
Description: Propagation of $[cAMP]_i$ waves in the loose mound and tight mound stages (see Fig. 1c and d for corresponding snapshots). Fluorescent images of Flamindo2 were acquired by an epifluorescence microscope at 20-second intervals for 20 minutes. Wave propagation is highlighted by the image subtraction of successive images (120-second intervals). Two concatenated movies show the different stages of the same developing mound. Left, loose mound stage. Right, tight mound stage. At the loose mound stage, $[cAMP]_i$ waves show rotational propagation in the mound. In the tight mound stage, $[cAMP]_i$ waves propagate from the top of the mound to the bottom. Scale bar, 50 μm.

File name: Supplementary Movie 3
Description: Wave propagation in streams flowing into an elongating slug (see Fig. 1e for corresponding snapshots). Fluorescent images were taken by an epifluorescence microscope at 30-second intervals for 20 minutes. Left, epifluorescent images of Flamindo2. Right, subtracted images showing the same mound. Wave propagation is highlighted by the image subtraction of successive images (90-second intervals). Scale bar, 100 μm.

File name: Supplementary Movie 4
Description: $[cAMP]_i$ monitoring through the developmental course of *Dictyostelium* cells by Flamindo2 (see Fig. 2a and b for corresponding snapshots and Flamindo2 signals). 3D images were taken by a confocal microscope at 30-second intervals. Data were obtained for 3–10 hours after starvation. The maximum projection of Z-stack fluorescent images is shown. Scale bar, 100 μm.

File name: Supplementary Movie 5

Description: External cAMP stimulation to an intact slug by the injection of cAMP into agar near the slug from a microcapillary (see Fig. 4c and d for corresponding snapshots and the Flamindo2 signals). Fluorescent images were taken by an epifluorescence microscope at 15-second intervals for 25.5 minutes. Left, DIC images. Right, fluorescent image of Flamindo2 (green) and diffusing TMR (magenta) mixed with cAMP to visualize the injected solution. Scale bar, 50 μm.

File name: Supplementary Movie 6
Description: Translocation of $PH_{Akt/PKB}$-GFP to the leading edge of cells in mounds and slugs (see Supplementary Fig. 8a–c for corresponding snapshots and $PH_{Akt/PKB}$-GFP signals). Fluorescent images were taken by a confocal microscope at 10-second intervals for 20 minutes. Left, a loose mound. Middle, a tight mound. Right, a slug. Scale bar, 50 μm.

File name: Supplementary Movie 7
Description: Aggregation of *acaA*-null cells expressing Flamindo2 in DB with exogenous cAMP pulses under microscopic observation (see Fig. 5a and d for corresponding snapshots and Flamindo2 signals). Images were taken by a confocal microscope at 1 minute intervals for 8 hours. Left, DIC images. Right, fluorescent images of Flamindo2. Scale bar, 100 μm.

File name: Supplementary Movie 8
Description: Slug formation of *acaA*-null cells expressing Flamindo2 on agar after cAMP pulse treatment (see Fig. 5b and e for corresponding snapshots and Flamindo2 signals in the first 3 hours). Images were taken by a confocal microscope at 1 minute intervals for 6 hours. Left, DIC images. Right, fluorescent images of Flamindo2. Scale bar, 100 μm.

File name: Supplementary Movie 9
Description: Simultaneous monitoring of $[cAMP]_i$ dynamics and cell differentiation during mound development (see Fig. 6a–c for corresponding snapshots and Flamindo2 and *ecmAO*::mRFPmars signals). 3D images were taken by a confocal microscope at 30-second intervals for 5 hours. The maximum projection of Z-stack fluorescent images is shown. Left, fluorescent images of Flamindo2. Right, fluorescent images of *ecmAO*::mRFPmars. Scale bar, 100 μm.

File name: Supplementary Movie 10
Description: Effect of caffeine on slug migration (see Fig. 7b and c for corresponding snapshots and quantitative analysis of the slug migration). Fluorescent images of Citrine were acquired by a confocal microscope at 30-second intervals for 30 minutes. The maximum projection of Z-stack fluorescent images of slugs is shown. Left, images of slugs with no treatment of caffeine (control). Right, images of slugs treated with 4 mM caffeine. Scale bar, 500 μm.

Description of Supplementary Data

File name: Supplementary Data 1
Description: The source data for the Figure 2.

File name: Supplementary Data 2
Description: The source data for the Figure 3.

File name: Supplementary Data 3
Description: The source data for the Figure 4.

File name: Supplementary Data 4
Description: The source data for the Figure 5.

File name: Supplementary Data 5
Description: The source data for the Figure 6.

File name: Supplementary Data 6
Description: The source data for the Figure 7.
